# New section and species in *Talaromyces*

**DOI:** 10.3897/mycokeys.68.52092

**Published:** 2020-07-07

**Authors:** Bing-Da Sun, Amanda J. Chen, Jos Houbraken, Jens C. Frisvad, Wen-Ping Wu, Hai-Lei Wei, Yu-Guang Zhou, Xian-Zhi Jiang, Robert A. Samson

**Affiliations:** 1 China General Microbiological Culture Collection Center, Institute of Microbiology, Chinese Academy of Sciences, Beijing 100101, China Institute of Microbiology Beijing China; 2 State Key Laboratory of Bioactive Substance and Function of Natural Medicines, Institute of Materia Medica, Chinese Academy of Medical Sciences and Peking Union Medical College, Beijing 100050, China Institute of Materia Medica Beijing China; 3 Microbiome Research Center, Moon (Guangzhou) Biotech Ltd., Guangzhou 510535, China Microbiome Research Center Guangzhou China; 4 Westerdijk Fungal Biodiversity Institute, Uppsalalaan 8, 3584 CT Utrecht, The Netherlands Westerdijk Fungal Biodiversity Institute Utrecht Netherlands; 5 Department of Biotechnology and Biomedicine, Technical University of Denmark, Kongens Lyngby, Denmark Technical University of Denmark Kongens Lyngby Denmark; 6 Novozymes China, No. 14, Xinxi Rd, Shangdi, Beijing, China Unaffiliated Beijing China; 7 Key Laboratory of Microbial Resources Collection and Preservation, Ministry of Agriculture, Institute of Agricultural Resources and Regional Planning, Chinese Academy of Agricultural Sciences, Beijing 100081, China Institute of Agricultural Resources and Regional Planning Beijing China

**Keywords:** Eurotiales, Penicillium resedanum, polyphasic taxonomy, section Tenues, soil

## Abstract

*Talaromyces* is a monophyletic genus containing seven sections. The number of species in *Talaromyces* grows rapidly due to reliable and complete sequence data contributed from all over the world. In this study agricultural soil samples from Fujiang, Guangdong, Jiangxi, Shandong, Tibet and Zhejiang provinces of China were collected and analyzed for fungal diversity. Based on a polyphasic approach including phylogenetic analysis of partial ITS, *BenA*, *CaM* and *RPB2* gene sequences, macro- and micro-morphological analyses, six of them could not be assigned to any described species, and one cannot be assigned to any known sections. Morphological characters as well as their phylogenetic relationship with other *Talaromyces* species are presented for these putative new species. *Penicillium resedanum* is combined in Talaromyces section Subinflati as *T. resedanus*.

## Introduction

The genus *Talaromyces* used to accommodate sexual *Penicillium* species (Benjamin 1955). The generic concept has changed in the last decade due to changes in nomenclatural rules and results of phylogenetic studies. Before 2011, various studies showed that asexual reproducing Penicillium species classified in subgenus Biverticillium and the sexual *Talaromyces* species form a monophyletic clade distinct from *Penicilliumsensu stricto* (LoBuglio et al. 1993; Berbee et al. 1995; Ogawa et al. 1997; Ogawa and Sugiyama 2000; Wang and Zhuang 2007; Houbraken and Samson 2011). In 2011, following the concepts of nomenclatural priority and single name nomenclature, Samson et al. (2011) transferred the majority of accepted Penicillium subgenus Biverticillium species to *Talaromyces*. A monograph of *Talaromyces* was provided based on a polyphasic species concept with seven sections *Bacillispori*, *Helici*, *Islandici*, *Purpurei*, *Subinflati*, *Talaromyces* and *Trachyspermi* (Yilmaz et al. 2014). This sectional classification was further supported by a four gene phylogeny (Chen et al. 2016). The number of species in *Talaromyces* grows rapidly due to reliable and complete sequence data contributed from all over the world (Visagie et al. 2015; Crous et al. 2016, 2017, 2018; Yilmaz et al. 2016a, b; Guevara-Suarez et al. 2017; Peterson and Jurjević 2017; Barbosa et al. 2018; Varriale et al. 2018; Rodríguez-Andrade et al. 2019; Rajeshkumar et al. 2019; Guevara-Suarez et al. 2020). It is noteworthy that in China many new species were discovered with another 19 new species reported (Chen et al. 2016; Wang QM et al. 2016; Wang XC et al. 2016, 2017; Jiang et al. 2018; Su and Niu 2018).

*Talaromyces* species are commonly distributed in a wide range of substrates, mostly in soil. Their main interest to food mycologists lies in their production of heat resistant ascospores and association with spoilage of pasteurized fruit juices and fruit-based products; the most commonly isolated heat resistant species include *T. bacilisporus*, *T. helicus*, *T. macrosporus*, *T. stipitatus* and *T. trachyspermus* (Dijksterhuis 2007; Pitt and Hocking 2009; Yilmaz et al. 2014). In addition, *T. flavus*, *T. funiculosus*, *T. pinophilus*, *T. purpurogenus*, *T. rugulosus* and *T. wortmannii* have been found quite frequently in food, including fruit, nuts and cereals (Pitt and Hocking 2009). *Talaromyces islandicus* can cause the yellowing of stored rice and has been reported from e.g. flour, peanuts, pecans, soybeans and maize (Saito et al. 1971; Sakai et al. 2005; Oh et al. 2008; Pitt and Hocking 2009).This species produces unique mycotoxins such as cyclochlorotine, islanditoxin, erythroskyrine and luteoskyrin, which are carcinogenic liver and kidney toxins (Uraguchi et al. 1961, 1972; Uraguchi 1962; Ueno and Ishikawa 1969; Bouhet et al. 1976; Stark et al. 1978). Other mycotoxins produced by *Talaromyces* members include rugulosin and skyrin (by members of sectionIslandici), botryodiploidin (*T. coalescens* and *T. stipitatus*), rubratoxin (*T. purpurogenus*), rugulovasine (*T. purpurogenus* and *T. wortmannii*) and secalonic acid D & F (*T. dendriticus*, *T. flavovirens*, *T. funiculosus*, *T. minioluteus*, *T. pseodostromaticus*, *T. siamensis* and *T. stipitatus*) (Yilmaz et al. 2014).

*Talaromyces* contains several species that are reported to cause infections in humans. *Talaromyces marneffei* has been exclusively associated with acquired immunodeficiency syndrome (AIDS) caused by human immunodeficiency virus (HIV) infections (Supparatpinyo et al. 1994; Limper et al. 2017). Other species like *T. indigoticus*, *T. helicus*, *T. piceus*, *T. purpurogenus*, *T. radicus*, *T. rugulosus* and *T. verruculosus* have been reported in superficial or disseminated, fatal infections (Horré et al. 2001; Santos et al. 2006; de Vos et al. 2009; Weisenborn et al. 2010; Tomlinson et al. 2011; de Hoog et al. 2014). Recently, four new members of *Talaromyces* were reported from clinical sources, and more studies are needed to complete the distribution and the relevance of these new fungi in human and animal disease (Guevara-Suarez et al. 2017).

On the other hand, species in *Talaromyces* are good producers of anticancer, antibacterial and antifungal compounds (Bladt et al. 2013; Zhai et al. 2016; Nicoletti et al. 2018), antiproliferative and antioxidative compounds (Kumari et al. 2018), enzymes (Isbelia et al. 1999; Narikawa et al. 2000; Pol et al. 2012; Maeda et al. 2013; Antonopoulou et al. 2018; Lian et al. 2018; Xu et al. 2018) and natural colourants (Frisvad et al. 2013; Zaccarim et al. 2018). Several species also proved to be effective biocontrol agents against soil-borne pathogens. *Talaromyces flavus* suppresses Verticillium wilt of tomato, eggplant and potato (Dutta et al. 1981; Marois et al. 1984; Fahima and Henis 1995), parasitizes *Sclerotinia sclerotiorum*, *Rhizoctonia solani* and *Sclerotium rolfsii* (Boosalis 1956; McLaren et al. 1986), degrades cell walls of *Pythium ultimum* and *Fusarium equisetii* (Inglis and Kawchuk 2002), shows antagonistic activities against *Cylindrocarpon destructans*, *Fusarium oxysporum*, *Rhizoctonia solani*, *Sclerotinia nivalis*, *Botrytis cinerea*, *Phytophthora capsici* and increases the dehiscence ratios of ginseng seed (Kim et al. 2017). *Talaromyces pinophilus* shows antagonistic activity and mycoparasitic behavior on *Rhizoctonia solani* and *Botrytis cinerea* (Alagesaboopathi 1994; Abdel-Rahim and Abo-Elyousr 2018) and shows plant growth-promoting effects on Waito-C rice (Khalmuratova et al. 2015).

In this study, we collected agricultural soil samples from Fujiang, Guangdong, Jiangxi, Shandong, Tibet and Zhejiang provinces in China. After isolation and identification, six *Talaromyces* species could not be assigned to any known species. A polyphasic approach including phylogenetic analysis of partial ITS, β-tubulin (*BenA*), calmodulin (*CaM*) and RNA polymerase II second largest subunit (*RPB2*) gene sequences and macro- and micro-morphological data were used to delimitate the new species and section in this genus.

## Materials and methods

### Isolates

Soil samples were collected from six provinces from China as mentioned above. A general dilution-plate method was used to isolate fungi, bacteria and actinomycetes. As for fungi, Potato Dextrose Agar (PDA, Guangdong huankai microbiological technology co., LTD) and Rose Bengal Medium (RBM, Beijing luqiao technology co., Ltd) with antibiotics (tetracycline hydrochloride and chloramphenicol with the final concentration of 100 mg/ml) were used. Obtained strains were purified and sub-cultured on malt extract agar (MEA, Guangdong huankai microbiological technology co., Ltd). Reference strains used in this study were obtained from the China General Microbiological Culture Collection Center (CGMCC), Beijing, China, the CBS culture collection and the working collection of the Applied and Industrial Mycology department (DTO), both housed at the Westerdijk Fungal Biodiversity Institute (Utrecht, the Netherlands). An overview of strains is listed in Table 1. For other strains used in the phylogenetic analyses, readers are referred to Chen et al. 2016; Crous et al. 2016, 2017, 2018; Wang QM et al. 2016; Wang XC et al. 2016, 2017; Yilmaz et al. 2016a, b; Guevara-Suarez et al. 2017; Peterson and Jurjević 2017; Barbosa et al. 2018; Jiang et al. 2018; Su and Niu 2018; Varriale et al. 2018; Rajeshkumar et al. 2019; Rodríguez-Andrade et al. 2019; Guevara-Suarez et al. 2020.

**Table 1.** *Talaromyces* strains used in this study.

Section	Species name	Strain no.	Substrate and origin	GenBank accession nr.			
				ITS	BenA	CaM	RPB2
* Talaromyces *	*Talaromyces brevis*	CBS 141833T= DTO 349-E7	Soil, Beijing, China	MN864269	MN863338	MN863315	MN863328
		DTO 307-C1	Soil, Zonguldak, Turkey	MN864270	MN863339	MN863316	MN863329
		CBS 118436 = DTO 004-D8	Soil, Maroc	MN864271	MN863340	MN863317	MN863330
	*Talaromyces rufus*	CBS 141834 T= DTO 349-D7 = CGMCC 3.13203	Soil, Yunnan, China	MN864272	MN863341	MN863318	MN863331
		DTO 274-C5	Soil, Korea	MN864273	MN863342	MN863319	n.a.
	*Talaromyces aspriconidius*	CBS 141835 T= DTO 340-F8	Soil, Yunnan, China	MN864274	MN863343	MN863320	MN863332
* Tenues *	*Talaromyces tenuis*	CBS 141840 T = DTO 340-G9	Soil, Guizhou, China	MN864275	MN863344	MN863321	MN863333
* Trachyspermi *	*Talaromyces albisclerotius*	CBS 141839 T = DTO 340-G5	Soil, Guizhou, China	MN864276	MN863345	MN863322	MN863334
* Subinflati *	*Talaromyces guizhouensis*	CBS 141837 T = DTO 340-G8	Soil, Guizhou, China	MN864277	MN863346	MN863323	MN863335
		DTO 054-C8	Soil from rainforest, Langkawi, Malaysia	MN864278	MN863347	MN863324	MN863336
		DTO 054-A7	Soil from rainforest, Langkawi, Malaysia	MN864279	MN863348	MN863325	MN863337
	*Talaromyces resedanus*	CBS 181.71T = DTO 376-A7 = ATCC 22356 = FRR 578 = IMI 062877 = NRRL 578	Soil, A1 horizon of Podzol, Victoria, Seychelles	MN864280	MN863349	MN863326	n.a.
		CBS 184.90 = DTO 376-A8 = UPSC 2879	Soil in greenhouse, Sweden	MN864281	MN863350	MN863327	n.a.

### DNA extraction, PCR amplification and sequencing

Strains were grown for 1 wk on MEA prior to DNA extraction. DNA was extracted using the Ultraclean TM Microbial DNA isolation Kit (MoBio, Solana Beach, U.S.A.) and stored at -20 °C. The ITS, *BenA*, *CaM*, and *RPB2* genes were amplified and sequenced using methods and primers previously described (Houbraken and Samson 2011; Yilmaz et al. 2014).

### Phylogenetic analysis

For sectional classification in *Talaromyces*, a four-gene phylogeny combining ITS, *BenA*, *CaM* and *RPB2* sequences was used. Prior to combining the datasets, single gene alignments were generated using MAFFT v. 7 (Katoh et al. 2019), and then trimmed at both ends. Aligned datasets were subsequently concatenated using Mesquite v 3.6 (Maddison and Maddison 2018). For each section, single gene phylogenies were generated to determine the phylogenetic relationship among species. The most suitable substitution model was determined using FindModel (Posada and Crandall 1998). Bayesian analyses were performed with MrBayes v. 3.2 (Ronquist et al. 2012). The sample frequency was set to 100 and the first 25% of trees were removed as burn-in. Maximum likelihood analyses including 1000 bootstrap replicates were run using RAxML (Kozlov et al. 2019). *Trichocoma paradoxa* (CBS 788.83^T^) was used as an outgroup in the *Talaromyces* phylogeny. Sequences of *T. trachyspermus* (CBS 373.48^T^), *T. dendriticus* (CBS 660.80^T^) and *T. purpurogenus* (CBS 286.36^T^) were used as outgroups in *Talaromyces* sections *Subinflati*, *Talaromyces* and *Trachyspermi*, respectively. The resulting trees were visualized with FigTree v1.4.2 and edited in Adobe Illustrator CS5. Bayesian inference (BI) posterior probabilities (pp) values and bootstrap (bs) values are labelled on nodes. Values less than 0.95 pp and 75% bs are not shown. Branches with posterior probability values of 1 and bootstrap values higher than 95% are thickened. Newly obtained sequences were deposited in GenBank.

### Morphological analysis

Macroscopic characters were studied on Czapek yeast autolysate agar (CYA), CYA supplemented with 5% NaCl (CYAS), yeast extract sucrose agar (YES), creatine sucrose agar (CREA), dichloran 18% glycerol agar (DG18), oatmeal agar (OA) and malt extract agar (MEA; Oxoid malt) (Samson et al. 2010). Isolates were inoculated at three points on 90 mm Petri dishes and incubated for 7 d at 25 °C in darkness. Additional CYA plates were incubated at 30 and 37 °C, and an additional MEA plate was incubated at 30 °C. After 7 d of incubation, colony diameters were recorded. The colony texture, degree of sporulation, obverse and reverse colony colors, the production of soluble pigments and exudates were noted. Acid production on CREA is indicated by a change in the pH sensitive bromocresol purple dye, from a purple to yellow color in media surrounding colonies. For ascoma production, OA, MEA and CYA plates were incubated for up to four wks. Color codes used in description refer to Rayner (1970).

Microscope preparations were made from 1 wk-old colonies grown on MEA. Production of ascomata, asci and ascospores was determined on 2–3 wk-old colonies on OA. Size of ascospores and conidia were measured without ornamentation. Lactic acid (60%) was used as mounting fluid and 96% ethanol was applied to remove the excess of conidia. A Zeiss Stereo Discovery V20 dissecting microscope and Zeiss AX10 Imager A2 light microscope equipped with Nikon DS-Ri2 cameras and software NIS-Elements D v4.50 were used to capture digital images.

## Results

### Phylogeny

**Figure 1.**
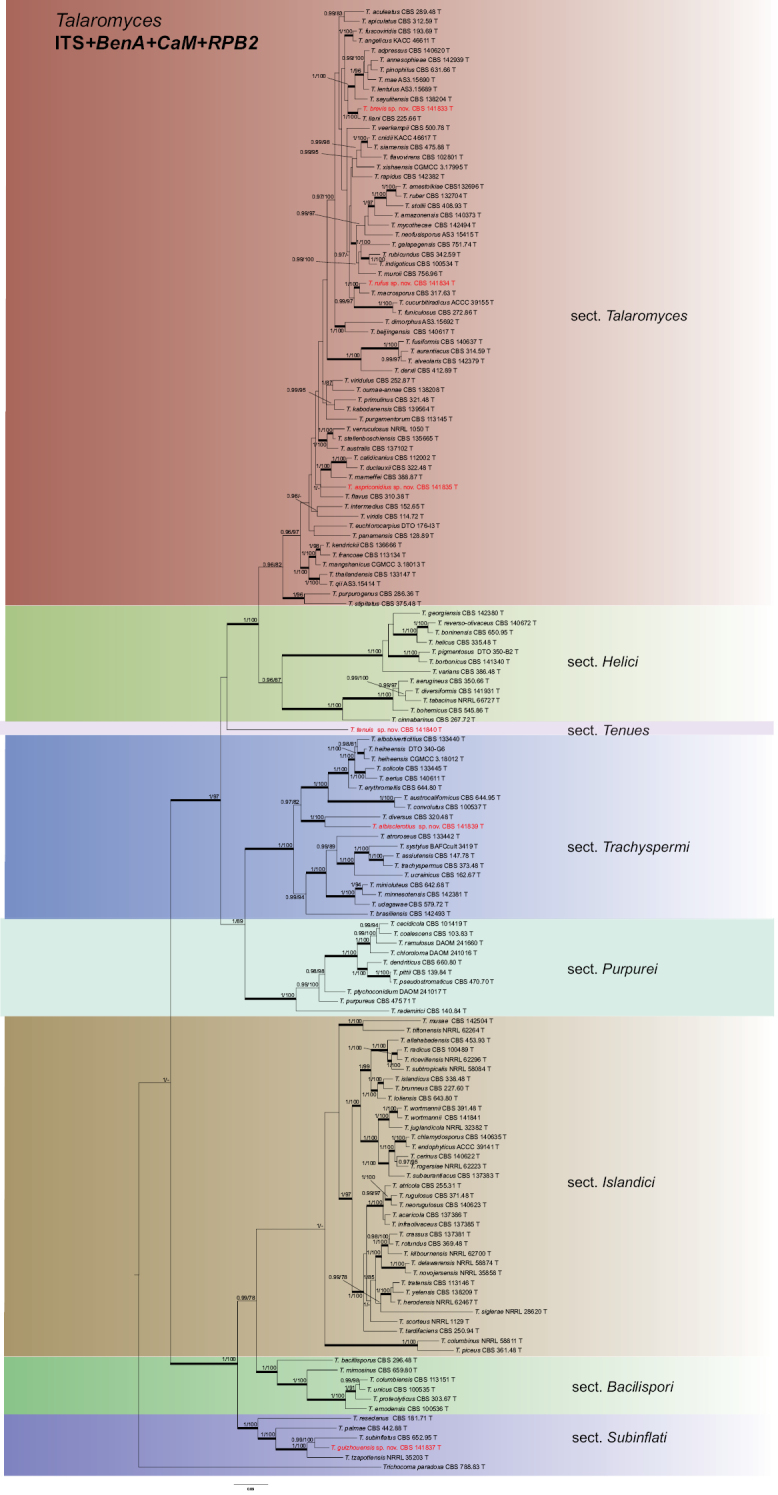
Concatenated phylogeny of the ITS, *BenA*, *CaM* and *RPB2* gene regions of species from *Talaromyces*. Branches with values more than 1 pp and 95% bs are thickened, supports lower than those values are indicated with a dash (-). *Trichocoma paradoxa* (CBS 788.83^T^) was chosen as outgroup. T: ex-type.

The individual ITS, *BenA*, *CaM* and *RPB2* datasets consist of 653, 591, 782 and 802 characters, respectively, and were combined to study the relationship within *Talaromyces*. The most optimal model for each dataset is listed in Table 2. Eight well-supported lineages are present in the multigene phylogenic analysis (Fig. 1). Seven lineages agree with sectional classification by Yilmaz et al. 2014 and one lineage, represented by a new species described here (*Talaromyces tenuis*), could not be assigned to any known section. This lineage is sister to sections *Talaromyces* and *Helici* but cannot be assigned into any of them. Based on its phylogenetic and morphological peculiarity (see description below), the lineage is described as a new section named *Tenues*. Furthermore, five new species are distributed over three sections, *T. brevis*, *T. rufus* and *T. aspriconidius* in section Talaromyces; *T. albisclerotius* in section Trachyspermi and *T. guizhouensis* in section Subinflati.

**Table 2.** Sequence data sets and models used in phylogeny.

Section	Sequence data sets							
	ITS (bp)	Substitution model	*BenA* (bp)	Substitution model	*CaM* (bp)	Substitution model	*RPB2* (bp)	Substitution model
Overview *Talaromyces*	653	GTR+G	591	K2P+G	782	GTR+G	802	GTR+G
section Subinflati	751	GTR+G	458	K2P+G	520	GTR+G	893	K2P+G
section Talaromyces	539	GTR+G	398	HKY+G	528	GTR+G	838	GTR+G
section Trachyspermi	505	GTR+G	422	GTR+G	556	GTR+G	852	GTR+G

**Figure 2.**
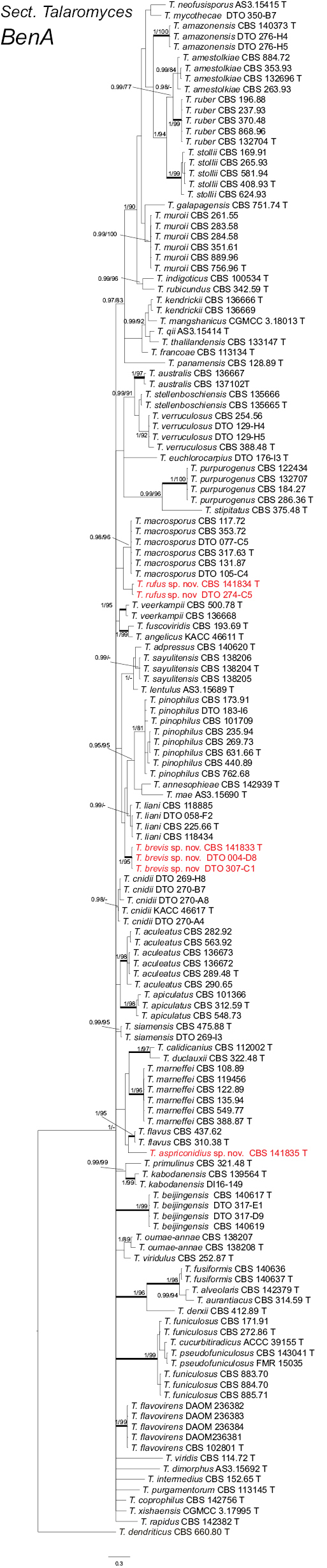
Phylogeny of *BenA* for species classified in Talaromyces section Talaromyces. Branches with values more than 1 pp and 95% bs are thickened, supports lower than those values are indicated with a dash (-). *Talaromyces dendriticus* (CBS 633.80^T^) was chosen as outgroup. T: ex-type.

In section Talaromyces, *T. rufus* and *T. aspriconidius* can be separated via each single gene phylogram. *Talaromyces rufus* is close to *T. macrosporus* based on *BenA*, *CaM* and *RPB2* phylograms and forms a separate lineage in ITS phylogram. *Talaromyces aspriconidius* is close to *T. primulinus* based on *RPB2* phylogram, but clusters with *T. flavus* based on *BenA* phylogram, and forms a separate lineage in *CaM* and ITS phylograms. *Talaromyces brevis* is closely related to *T. liani*, it can be differentiated via *BenA*, *CaM* and *RPB2* phylograms, but not via ITS phylogram (Fig. 2; Suppl. materials: 1–3).

**Figure 3.**
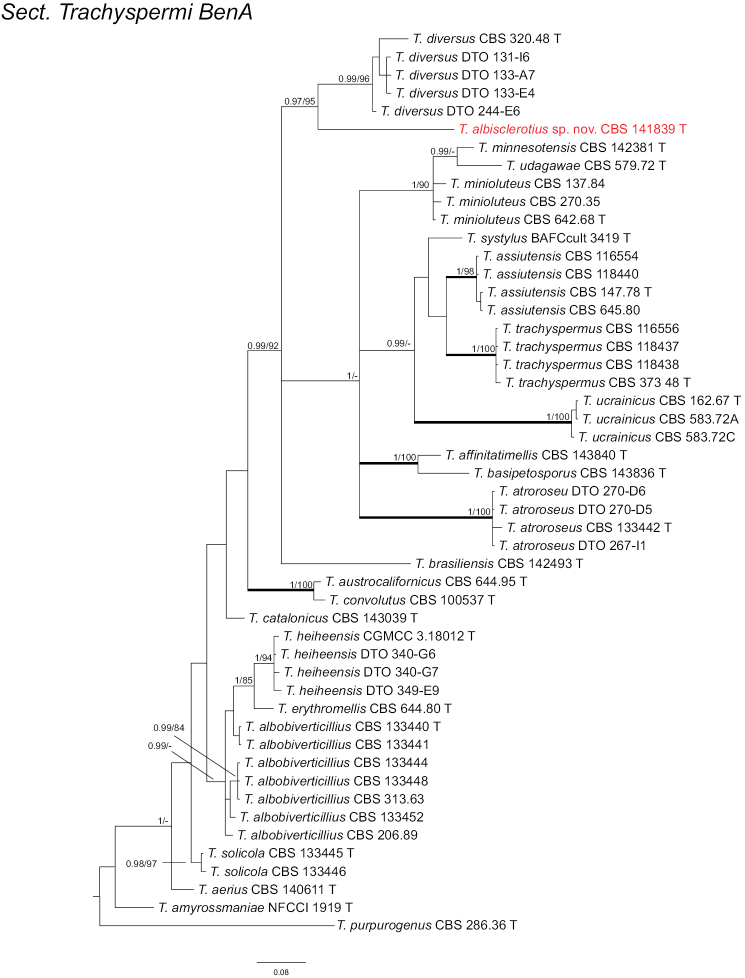
Phylogeny of *BenA* for species classified in Talaromyces section Trachyspermi. Branches with values more than 1 pp and 95% bs are thickened, supports lower than those values are indicated with a dash (-). *Talaromyces purpurogenus* (CBS 286.36^T^) was chosen as outgroup. T: ex-type.

In section Trachyspermi, *T. albisclerotius* can be well-separated in four single phylograms; this species clusters with *T. diversus* in *BenA*, *CaM* and *RPB2* phylograms, and forms a separate lineage in ITS phylogram (Fig. 3; Suppl. materials: 4–6).

**Figure 4.**
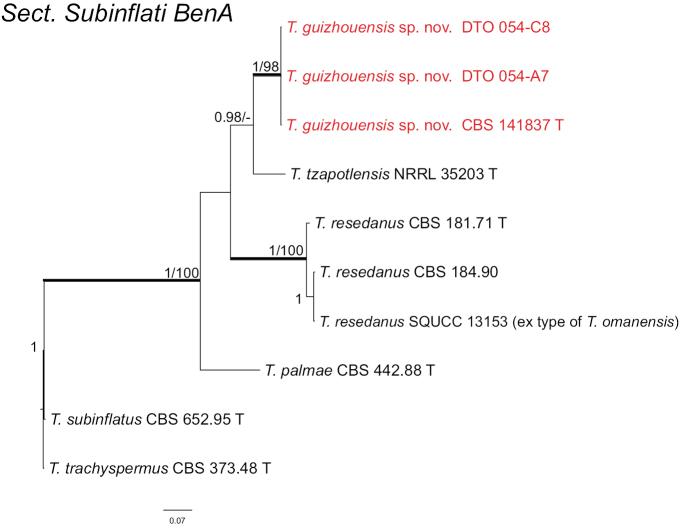
Phylogeny of *BenA* for species classified in Talaromyces section Subinflati. Branches with values more than 1 pp and 95% bs are thickened, supports lower than those values are indicated with a dash (-). *Talaromyces trachyspermus* (CBS 373.48^T^) was chosen as outgroup. T: ex-type.

*Talaromyces guizhouensis* is assigned in section Subinflati and *P. resedanum* also belongs to this section according to our multigene analysis. With the newly described *T. tzapotlensis* and *T. omanensis* the total number of taxa belonging to section Subinflati increased from two to five since it was established in 2014. *Talaromyces omanensis* shares same ITS, *BenA* and *CaM* sequences with *T. resedanus* CBS 184.90. *Talaromyces guizhouensis* is close to *T. tzapotlensis* and *T. subinflatus* in each single gene phylogram (Fig. 4, Suppl. materials: 7–9).

### Identification

The five new species *T. albisclerotius*, *T. aspriconidius*, *T. guizhouensis*, *T. rufus*, *T. tenuis* can be identified by ITS, *BenA*, *CaM* and *RPB2* sequences. *Talaromyces brevis* cannot be separated from *T. liani* (strains CBS 225.66^T^, CBS 118885, CBS 118434 and DTO 058-F2) by its ITS sequence, but it can be differentiated from *T. liani* by *BenA* (97.3% similarity, 366/376 bp), *CaM* (99.5% similarity, 463/465 bp) and *RPB2* (99% similarity, 838/846 bp).

## Taxonomy

### Talaromyces section Tenues

Taxon classificationFungiEurotialesAspergillaceae

B.D. Sun, A.J. Chen, Houbraken & Samson sect. nov.

6770C3CD-40B6-57FB-A11D-00B30DAF2128

833138

#### Typus.

*Talaromyces tenuis* B.D. Sun, A.J. Chen, Houbraken & Samson

#### Description.

Conidiophores monoverticillate or biverticillate, with hyaline, thin stipes, colonies grow restrictedly on CYA, YES, DG18, slightly faster on MEA and OA, no growth on CYAS and CREA at 25 °C and CYA incubated at 37 °C.

Phylogenetic analysis places Talaromyces section Tenues sister to sections *Talaromyces* and *Helici* (Fig. 1); however, statistical support for this relationship is lacking. Using a nine-gene sequence data set, Houbraken et al. (2020) confidently shows that *Talaromyces* sp. CBS 141840 (= *T. tenuis*, the sole representative of the section) is sister to sect. Purpurei and *Trachyspermi*. section Trachyspermi species produce abundant red pigments (Yilmaz et al. 2014), while *Talaromyces tenuis* does not. section Purpurei species generally grow rapidly on CYA and MEA, and usually produce synnemata after two to three weeks of incubation (Yilmaz et al. 2014).

#### Etymology.

Named after the type species of the section, *Talaromyces tenuis*.

### Talaromyces tenuis

Taxon classificationFungiEurotialesAspergillaceae

B.D. Sun, A.J. Chen, Houbraken & Samson sp. nov.

84420C8A-B025-53A1-808E-5777DBFB18A0

833136

Fig. 5

#### Typus.

**China**, Guizhou, soil, 2014, isolated by X.Z. Jiang, Holotype CBS H-22838, culture ex-holotype CBS 141840 = DTO 340-G9.

#### ITS barcode.

MN864275. Alternative identification markers: *BenA* = MN863344, *CaM* = MN863321, *RPB2* = MN863333.

#### Diagnosis.

*Talaromyces tenuis* produces hyaline, thin conidiophores, yellow mycelium on MEA and OA, and grows very restrictedly on CYA, YES and DG18.

#### In.

**Talaromyces section Tenues**

#### Colony diam, 7 d (mm).

CYA 7–8; CYA 30 °C 5–8; CYA 37 °C No growth; MEA 18–20; MEA 30 °C 10–11; OA 12–14; YES 9–10; CREA No growth; CYAS No growth; DG18 2–3.

#### Colony characters.

CYA 25 °C, 7 d: Colonies moderately deep, plane; margins entire; mycelium white; texture floccose; sporulation absent; soluble pigments absent; exudates absent; reverse white. MEA 25 °C, 7 d: Colonies moderately deep, plane; margins entire; mycelium sulphur yellow (15) or ochreous (44); texture floccose; sporulation sparse; conidia *en masse* white or greyish yellow-green (68); soluble pigments absent; exudates absent; reverse ochreous (44) to umber (9). YES 25 °C, 7 d: Colonies moderately deep, plane; margins entire; mycelium white; texture floccose; sporulation absent; soluble pigments absent; exudates absent; reverse buff (45). DG18 25 °C, 7 d: Colonies moderately deep, plane; margins entire; mycelium white; texture floccose; sporulation absent; soluble pigments absent; exudates absent; reverse white. OA 25 °C, 7 d: Colonies moderately deep, plane; margins entire; mycelium pale luteous (11); texture floccose; sporulation absent; soluble pigments absent; exudates absent; reverse buff (45).

**Figure 5.**
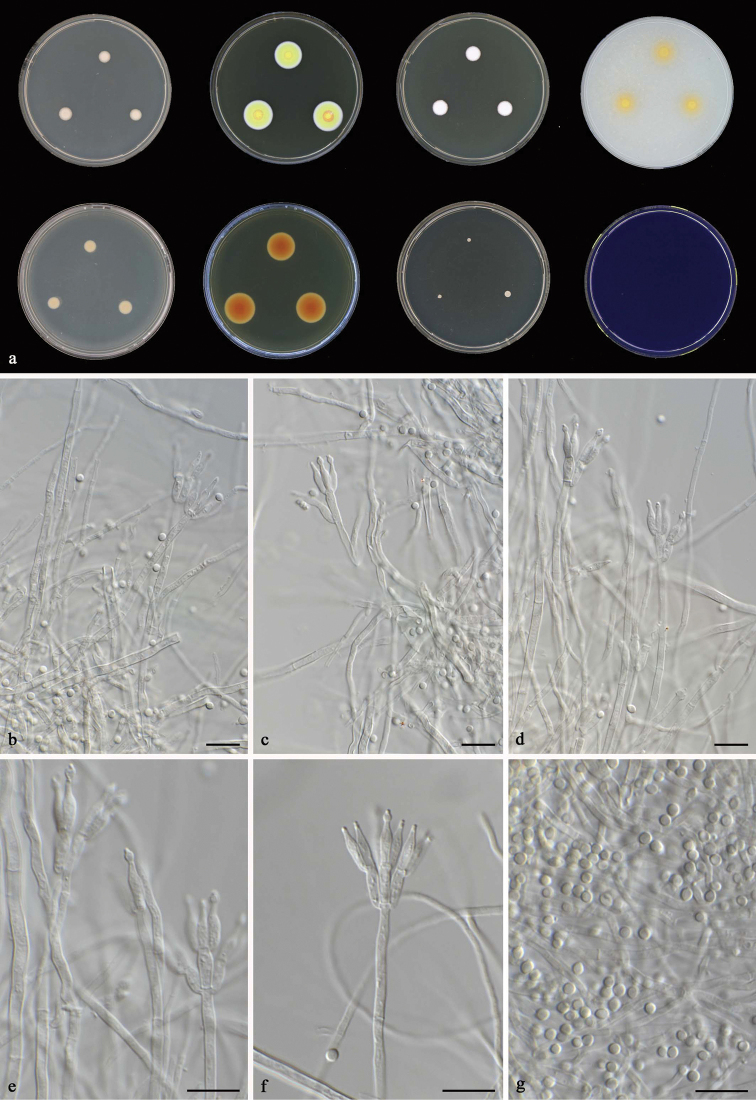
*Talaromyces tenuis* CBS 141840^T^**a** colonies from left to right (top row) CYA, MEA, YES and OA; (bottom row) CYA reverse, MEA reverse, DG 18 and CREA**b–g** conidiophores and conidia. Scale bars: 10 μm (**b–g**).

#### Micromorphology.

Conidiophores monoverticillate or biverticillate, stipes smooth, 80–150 × 2–3 μm; metulae 2–3, divergent, 8–9 × 2–2.5 μm; phialides 2–3, acerose, 8–9.5 × 2–2.5 μm; conidia smooth, globose to subglobose, 2–3 × 2–2.5 μm. Ascomata not observed.

#### Note.

*Talaromyces tenuis* is phylogenetically distinct and is basal to species belonging to sections *Talaromyces* and *Helici* (Fig. 1). In a nine-gene phylogeny, it is sister to sections *Purpurei* and *Trachyspermi* (Houbraken et al. 2020). This species is characterized by hyaline, thin conidiophores, and grows very restrictedly on CYA, YES and DG18; colonies on MEA and OA have prominent yellow mycelia.

#### Etymology.

Latin, *tenuis*, refers to its thin conidiophores.

### Talaromyces albisclerotius

Taxon classificationFungiEurotialesAspergillaceae

B.D. Sun, A.J. Chen, Houbraken & Samson sp. nov.

6F3414A0-09C1-57DE-B5A0-A1BC48EF98A4

833135

Fig. 6

#### Typus.

**China**, Xinjiang, soil, 2002, isolated by L. Cai, Holotype CBS H-22837, culture ex-holotype CBS 141839 = DTO 340-G5.

#### ITS barcode.

MN864276. Alternative identification markers: *BenA* = MN863345, *CaM* = MN863322, *RPB2* = MN863334.

#### Diagnosis.

*Talaromyces albisclerotius* produces white sclerotia on OA, grows restrictedly on CYA, YES, DG18 and OA and does not grow on CYAS.

#### In.

**Talaromyces section Trachyspermi**

#### Colony diam, 7 d (mm).

CYA 5–8; CYA 30 °C 3–4; CYA 37 °C No growth; MEA 19–20; MEA 30 °C 8–9; OA 13–14; YES 6–7; CREA No growth; CYAS No growth; DG18 5–6.

#### Colony characters.

CYA 25 °C, 7 d: Colonies moderately deep, slight sulcate; margins entire; mycelium white; texture floccose; sporulation dense; conidia *en masse* greyish yellow-green (68); soluble pigments absent; exudates absent; reverse buff (45). MEA 25 °C, 7 d: Colonies moderately deep, sulcate; margins entire; mycelium white and primrose (66); texture floccose; sporulation dense; conidia *en masse* pistachio green (92); soluble pigments absent; exudates absent; reverse ochreous (44). YES 25 °C, 7 d: Colonies moderately deep, plane; margins entire; mycelium white; texture floccose; sporulation moderately dense; conidia *en masse* pistachio green (92); soluble pigments absent; exudates absent; reverse buff (45). DG18 25 °C, 7 d: Colonies moderately deep, plane; margins entire; mycelium sulphur yellow (15); texture floccose; sporulation sparse; conidia *en masse* greyish yellow-green (68); soluble pigments absent; exudates absent; reverse sulphur yellow (15). OA 25 °C, 7 d: Colonies moderately deep, plane; margins entire; mycelium white and primrose (66); texture velvety; sporulation dense; conidia *en masse* yellow green (71); soluble pigments absent; exudates clear droplets; reverse greyish yellow-green (68). CREA 25 °C, 7 d: No growth.

#### Micromorphology.

Conidiophores biverticillate, with a minor proportion having subterminal branches; stipes smooth, 70–130 × 3–4 μm, extra branches 10–20 μm; metulae 3–5, divergent, 8.5–11 × 4–4.5 μm; phialides 4–6, acerose, 9–11 × 3–5 μm; conidia smooth, subglobose to fusiform, 2–4.5× 3–4 μm. Ascomata not observed, white sclerotia present on OA after 1 wk.

#### Notes.

*Talaromyces albisclerotius* is characterized by the production of white sclerotia on OA after 1 wk incubation; these sclerotia remain sterile and no ascospores are observed after prolonged incubation up to eight wk. *Talaromyces assiutensis* and *T. trachyspermus* could produce white ascomata, but their ascomata mature after weeks and release ascospores (Yilmaz et al. 2014). Phylogenetically, *T. albisclerotius* clusters with *T. diversus* and *T. brasiliensis*, but *T. diversus* grows faster on MEA, and *T. brasiliensis* produces rough conidia (Yilmaz et al. 2014; Barbosa et al. 2018).

#### Etymology.

Latin, *albisclerotius*, refers to its white sclerotia produced on OA.

**Figure 6.**
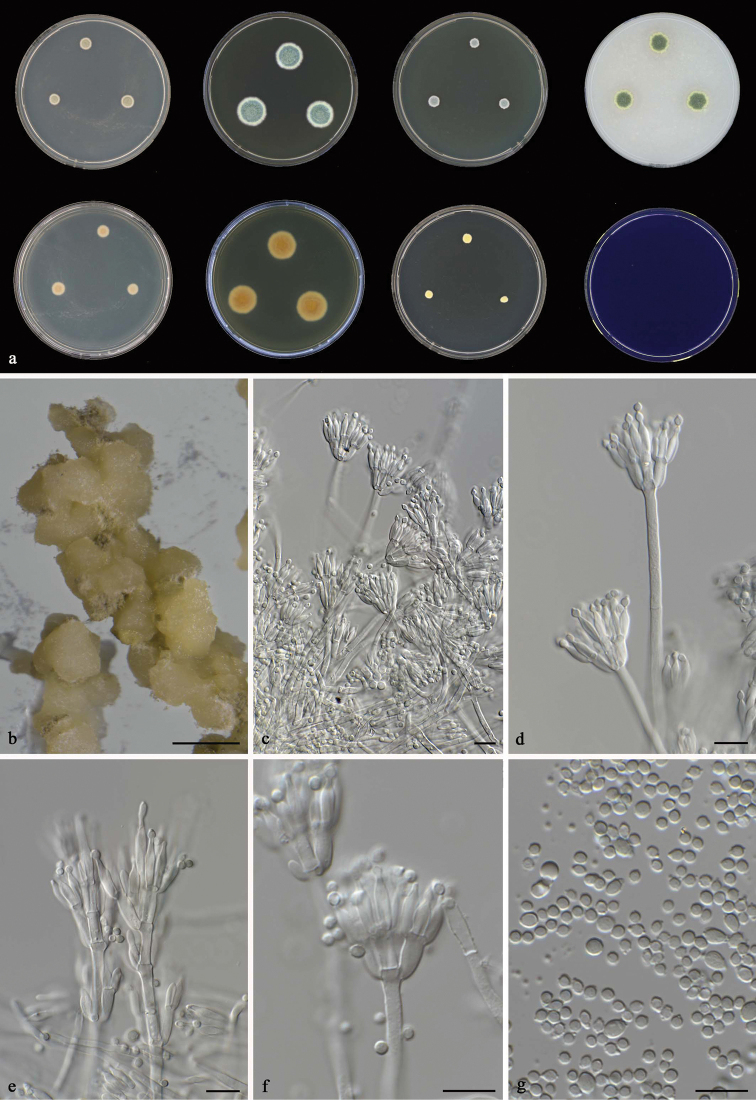
*Talaromyces albisclerotius* CBS 141839^T^**a** colonies from left to right (top row) CYA, MEA, YES and OA; (bottom row) CYA reverse, MEA reverse, DG 18 and CREA**b** sclerotia on OA after two weeks **c–g** conidiophores and conidia. Scale bars: 1000 μm (**b**), 10 μm (**c–g**).

### Talaromyces aspriconidius

Taxon classificationFungiEurotialesAspergillaceae

B.D. Sun, A.J. Chen, Houbraken & Samson sp. nov

BAA53332-CBC6-523E-8EF3-808AE135CCC9

833134

Fig. 7

#### Typus.

**China**, Yunnan, soil, 2008, isolated by L. Cai, Holotype CBS H-22833, culture ex-holotype CBS 141835 = DTO 340-F8.

#### ITS barcode.

MN864274. Alternative identification markers: *BenA* = MN863343, *CaM* = MN863320, *RPB2* = MN863332.

#### Diagnosis.

*Talaromyces aspriconidius* produces strikingly roughened, globose conidia, grows moderately on CYA and CYA at 30 °C, reaches 22–23 mm and 25–26 mm after 7 d.

#### In.

**Talaromyces section Talaromyces**

#### Colony diam, 7 d (mm).

CYA 22–23; CYA 30 °C 25–26; CYA 37 °C 22–23; MEA 36–37; MEA 30 °C 44–45; OA 38–42; YES 28–29; CREA 7–8; CYAS No growth; DG18 10–11.

#### Colony characters.

CYA 25 °C, 7 d: Colonies moderately deep, plane; margins entire; mycelium white and peach (4); texture floccose; sporulation moderately dense; conidia *en masse* greyish yellow-green (68); soluble pigments absent; exudates absent; reverse buff (45). MEA 25 °C, 7 d: Colonies moderately deep, plane; margins entire; mycelium white; texture floccose; sporulation moderately dense; conidia *en masse* leek green (49) and greyish yellow-green (68); soluble pigments absent; exudates brown droplets; reverse saffron (10). YES 25 °C, 7 d: Colonies moderately deep, plane; margins entire; mycelium white and peach (4); texture floccose; sporulation moderately dense; conidia *en masse* greyish yellow-green (68); soluble pigments absent; exudates absent; reverse saffron (10). DG18 25 °C, 7 d: Colonies moderately deep, plane; margins entire; mycelium white and primrose (66); texture floccose; sporulation dense; conidia *en masse* honey (64); soluble pigments absent; exudates absent; reverse greyish yellow-green (68). OA 25 °C, 7 d: Colonies moderately deep, plane; margins entire; mycelium white and primrose (66); texture floccose; sporulation dense; conidia *en masse* yellow-green (71); soluble pigments absent; exudates clear droplets; reverse greyish yellow-green (68). CREA 25 °C, 7 d: Moderate growth, acid production absent.

#### Micromorphology.

Conidiophores biverticillate, stipes smooth, 150–250 × 3–4 μm, metulae 4–5, divergent, 10–12 × 3–3.5 μm; phialides 4–6, acerose to flask shaped, 8–10.5 × 3–3.5 μm; conidia strikingly roughened, globose, 3–4 μm. Ascomata not observed.

#### Notes.

*Talaromyces aspriconidius* is characterized by its strikingly roughened, globose conidia. *Talaromyces aculeatus*, *T. apiculatus*, *T. diversus*, *T. solicola* and *T. verruculosus* also produce this kind of conidia. However, *T. aspriconidius* grows slower than *T. aculeatus*, *T. apiculatus* and *T. verruculosus*, and faster than *T. diversus* and *T. solicola* on CYA and CYA at 30 °C (Yilmaz et al. 2014).

#### Etymology.

Latin, *aspriconidius*, refers to its strikingly roughened conidia.

**Figure 7.**
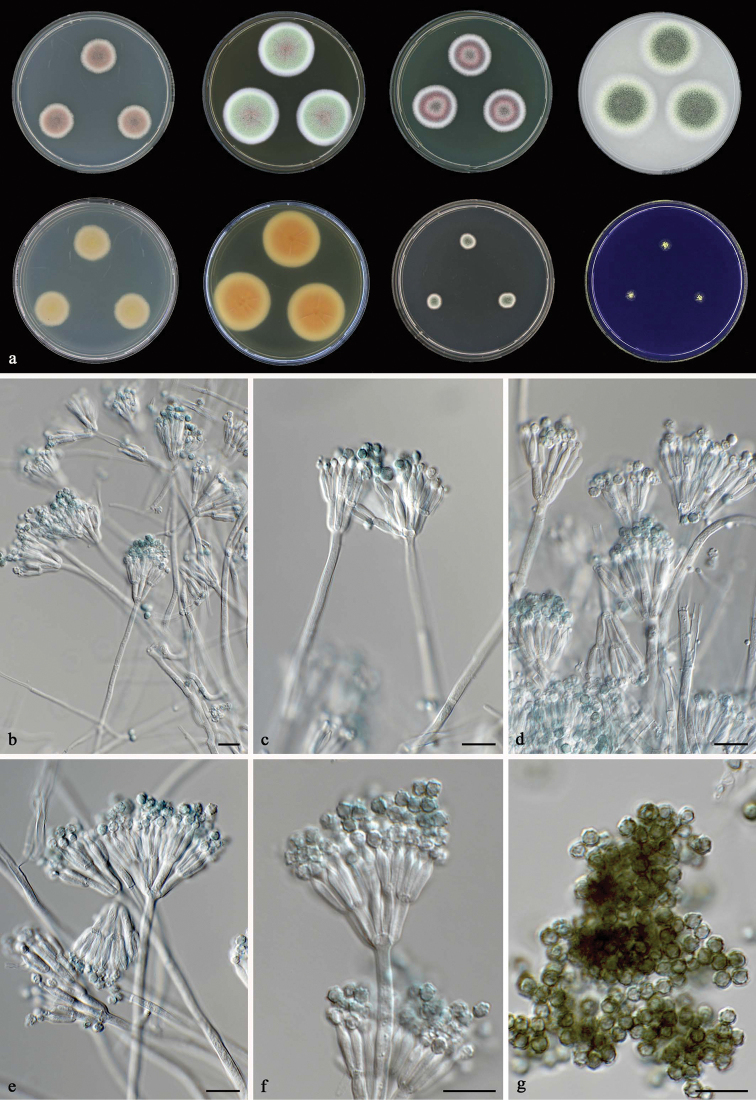
*Talaromyces aspriconidius* CBS 141835^T^**a** colonies from left to right (top row) CYA, MEA, YES and OA; (bottom row) CYA reverse, MEA reverse, DG 18 and CREA**b–g** conidiophores and conidia. Scale bars: 10 μm (**b–g**).

### Talaromyces brevis

Taxon classificationFungiEurotialesAspergillaceae

B.D. Sun, A.J. Chen, Houbraken & Samson sp. nov.

D94B7794-43E2-58E9-910F-B836D4CB60DE

833132

Fig. 8

#### Typus.

**China**, Beijing, soil, 2010, isolated by B.D. Sun, Holotype CBS H-22831, culture ex-holotype CBS 141833= DTO 349-E7.

#### Additional material examined.

**Turkey**, Zonguldak, soil, 2014, isolated by Rasime Demirel, culture DTO 307-C1. Maroc, soil, 2005, isolated by J. Dijksterhuis, culture CBS 118436 = DTO 004-D8.

#### ITS barcode.

MN864269. Alternative identification markers: *BenA* = MN863338, *CaM* = MN863315, *RPB2* = MN863328.

#### Diagnosis.

*Talaromyces brevis* produces short conidiophores measuring 15–50 × 3–4 μm, yellow to orange ascomata on OA and spiny ascospores measuring 3.5–4.5 × 3–4 μm.

#### In.

**Talaromyces section Talaromyces**

#### Colony diam, 7 d (mm).

CYA 30–31; CYA 30 °C 28–30; CYA 37 °C 25–26; MEA 50–51; MEA 30 °C 57–60; OA 39–43; YES 42–43; CREA 13–14; CYAS No growth; DG18 13–15.

#### Colony characters.

CYA 25 °C, 7 d: Colonies moderately deep, sulcate; margins entire; mycelium white and flesh (37); texture floccose; sporulation sparse; conidia *en masse* white; soluble pigments absent; exudates absent; reverse ochreous (44). MEA 25 °C, 7 d: Colonies moderately deep, plane; margins entire; mycelium white and primrose (66); texture floccose; sporulation sparse; conidia *en masse* white to greyish yellow-green (68); soluble pigments absent; exudates absent; reverse ochreous (44). YES 25 °C, 7 d: Colonies moderately deep, sulcate; margins entire; mycelium white; texture floccose; sporulation absent; soluble pigments absent; exudates absent; reverse ochreous (44). DG18 25 °C, 7 d: Colonies moderately deep, slightly raised at center, plane; margins entire; mycelium white; texture floccose; sporulation moderately dense; conidia *en masse* yellow-green (71); soluble pigments absent; exudates absent; reverse greyish yellow-green (68). OA 25 °C, 7 d: Colonies moderately deep, plane; margins entire; mycelium primrose (66); texture floccose; sporulation absent; soluble pigments absent; exudates absent; reverse primrose (66). Ascomata present. CREA 25 °C, 7 d: Moderate growth, acid production present.

#### Micromorphology.

Conidiophores monoverticillate and biverticillate; stipes smooth, 15–50 × 3–4 μm; metulae 3–5, divergent, 10–15 × 2.5–3 μm; phialides 4–6, flask-shaped, 9–13 × 2–4 μm; conidia smooth, subglobose to fusiform, 3–4(–5) × 2.5–3.5(–4.5) μm. Ascomata maturing after 2–3 wk of incubation on OA, yellow to orange, globose to subglobose, 400–550 μm; ascospores ellipsoidal, spiny, 3.5–4.5 × 3–4 μm.

#### Notes.

*Talaromyces brevis* is morphologically and phylogenetically close to *T. liani*, but the latter produces larger ascospores measuring 4–6 × 2.5–4 μm and does not produce acid on CREA (except *T. liani* CBS 118885 produces very weak acid) (Yilmaz et al. 2014).

#### Etymology.

Latin, *brevis*, refers to its short conidiophores.

**Figure 8.**
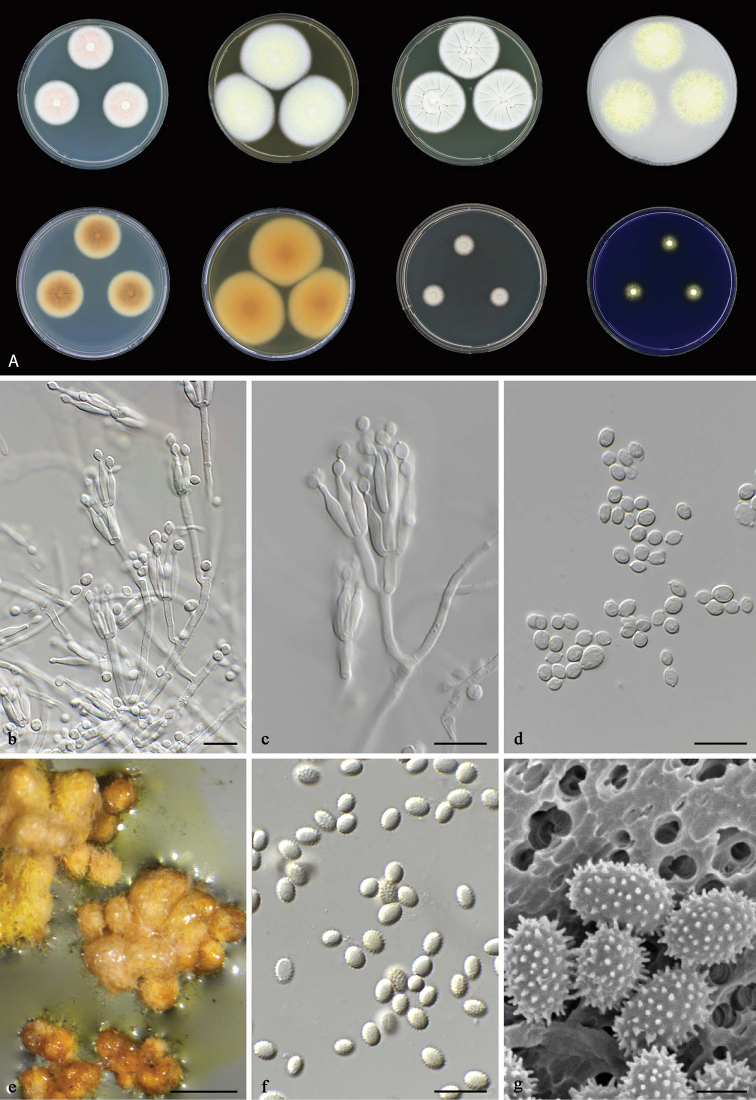
*Talaromyces brevis* CBS 141833^T^**a** colonies from left to right (top row) CYA, MEA, YES and OA; (bottom row) CYA reverse, MEA reverse, DG 18 and CREA**b–d** conidiophores and conidia **e** ascomata on OA after two weeks **f–g** ascospores. Scale bars: 10 μm (**b–d, f**), 1000 μm (**e**), 2 μm (**g**).

### Talaromyces guizhouensis

Taxon classificationFungiEurotialesAspergillaceae

B.D. Sun, A.J. Chen, Houbraken & Samson sp. nov.

4B355123-B815-57DF-88FF-E4ED77BC6C39

833131

Fig. 9

#### Typus.

**China**, Guizhou, soil, 2014, isolated by X.Z. Jiang, Holotype CBS H-22835, culture ex-holotype CBS 141837= DTO 340-G8.

#### Additional material examined.

**Malaysia**, Langkawi, soil from rainforest, 2007, isolated by J. Houbraken, culture DTO 054-C8. Malaysia, Langkawi, soil from rainforest, 2007, isolated by J. Houbraken, culture DTO 054-A7.

#### ITS barcode.

MN864277. Alternative identification markers: *BenA* = MN863346, *CaM* = MN863323, *RPB2* = MN863335.

#### Diagnosis.

*Talaromyces guizhouensis* grows poorly on CREA and DG18, does not produce synnemata as well as ascospores.

#### In.

**Talaromyces section Subinflati**

#### Colony diam, 7 d (mm).

CYA 8–9; CYA 30 °C 10; CYA 37 °C No growth; MEA 24–27; MEA 30 °C 18–19; OA 27–29; YES 12–13; CREA 2–3; CYAS No growth; DG18 4–5.

#### Colony characters.

CYA 25 °C, 7 d: Colonies moderately deep, plane; margins entire; mycelium white; texture floccose; sporulation absent; soluble pigments absent; exudates clear droplets; reverse saffron (10). MEA 25 °C, 7 d: Colonies moderately deep, raised at center, plane; margins entire; mycelium white; texture floccose; sporulation moderately dense; conidia *en masse* pistachio green (92); soluble pigments absent; exudates absent; reverse saffron (10). YES 25 °C, 7 d: Colonies moderately deep, raised at center, plane; margins entire; mycelium white; texture floccose; sporulation absent; soluble pigments absent; exudates clear droplets; reverse cream white. DG18 25 °C, 7 d: Colonies moderately deep, plane; margins entire; mycelium white; texture floccose; sporulation absent; soluble pigments absent; exudates absent; reverse cream white. OA 25 °C, 7 d: Colonies moderately deep, raised at center, plane; margins entire; mycelium white; texture floccose; sporulation moderately dense; conidia *en masse* pistachio green (92); soluble pigments absent; exudates clear droplets; reverse greyish lavender (98) at center, fading into saffron (10). CREA 25 °C, 7 d: Poor growth, acid production absent.

#### Micromorphology.

Conidiophores biverticillate, stipes smooth to finely rough, 150–300 × 3–4.5 μm, metulae 3–5, divergent, 11–13 × 3–5 μm; phialides 3–5, acerose to flask shaped, 9–10 × 3–3.5 μm; conidia finely rough, subglobose to fusiform, 2.5–4.5 × 2.5–3 μm. Ascomata not observed.

#### Notes.

section Subinflati previously contained two species namely *T. subinflatus* and *T. palmae*. These species do not resemble each other, although both grow poorly on CREA and DG18 (Yilmaz et al. 2014). *Talaromyces tzapotlensis* was included more recently (Peterson and Jurjević 2017) and we here expand this section with *T. guizhouensis* and *T. resedanus*. Like the other species in this section, *T. guizhouensis* also grows poorly on CREA and DG18. This species is phylogenetically related to *T. subinflatus*, but the latter grows very restrictedly on common media except MEA (Yilmaz et al. 2014). *Talaromyces palmae* produces indeterminate synnemata and short stipes (up to 85 μm) (Yilmaz et al. 2014) and these are not observed in *T. guizhouensis*. Furthermore, *T. tzapotlensis* grows faster on most media (e.g., 29–30 *vs* 8–9 mm on CYA; 10–11 *vs* 4–5 mm on DG18; 20–22 *vs* 2–3 mm on CREA, all diam. after 7 days (Peterson and Jurjević 2017) and *T. resedanus* does not grow on CREA and produces smaller conidia measuring 2–3 × 1.5–2 μm.

#### Etymology.

Latin, *guizhouensis*, refers to its origin, isolated from Guizhou, China.

**Figure 9.**
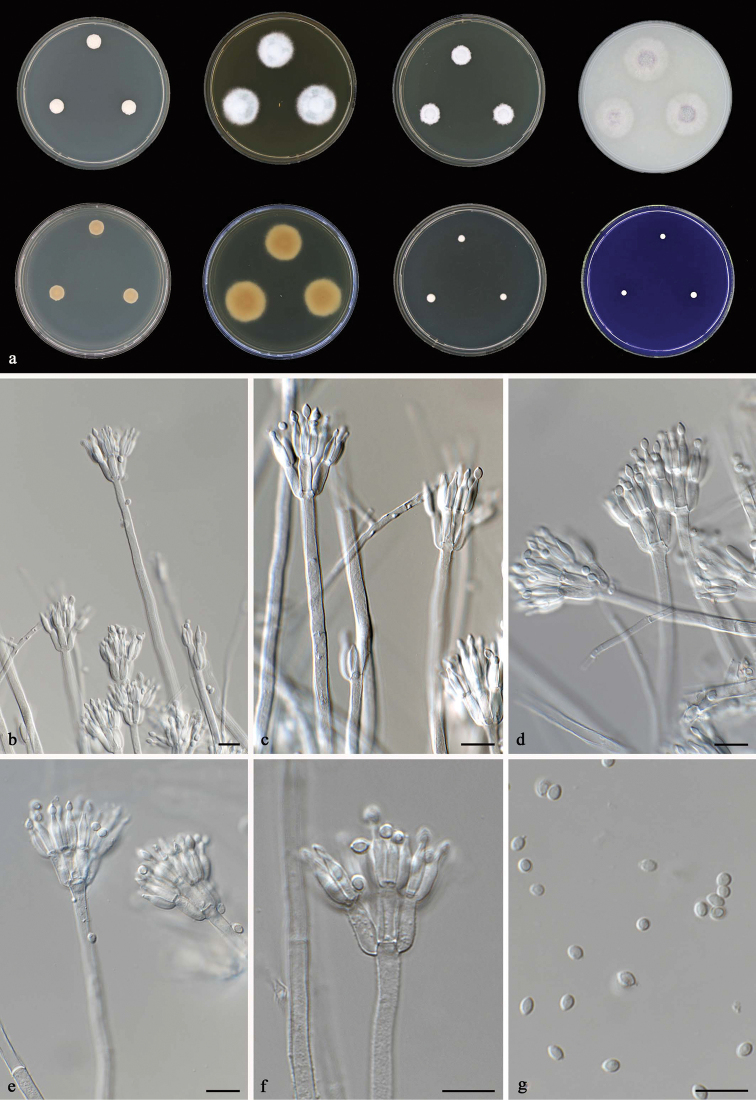
*Talaromyces guizhouensis* CBS 141837^T^**a** colonies from left to right (top row) CYA, MEA, YES and OA; (bottom row) CYA reverse, MEA reverse, DG 18 and CREA**b–g** conidiophores and conidia. Scale bars: 10 μm (**b–g**).

### Talaromyces resedanus

Taxon classificationFungiEurotialesAspergillaceae

(McLennan and Ducker) A.J. Chen, Houbraken & Samson comb. nov.

71CF7A9A-5641-5A52-B1CF-4A720AC657EF

302422

Fig. 10

Penicillium resedanum McLennan and Ducker, Aust. J. Bot. 2: 360. 1954. Basionym.
= Talaromyces omanensis Halo, Maharachch., Al-Yahyai and Al-Sadi, Phytotaxa 404: 192. 2019.

#### Typus.

**Australia**, Frankston in solo arenoso acido, Holotype IMI 062877, culture ex-holotype CBS 181.71 = DTO 376-A7 = ATCC 22356 = FRR 578 = IMI 062877 = NRRL 578.

#### Additional material examined.

**Sweden**, soil in greenhouse, 1989, isolated by O. Constantinescu, culture CBS 184.90 = DTO 376-A8 = UPSC 2879.

#### ITS barcode.

MN864280. Alternative identification markers: *BenA* = MN863349, *CaM* = MN863326, *RPB2* = MN969214.

#### In.

**Talaromyces section Subinflati**

#### Colony diam, 7 d (mm).

CYA 19–21; CYA 30 °C 18–20; CYA 37 °C 8–11; MEA 23–25; MEA 30 °C 15–21; OA 26–28; YES 17–19; CREA No growth; CYAS 5–7; DG18 8–9.

#### Colony characters.

CYA 25 °C, 7 d: Colonies moderately deep, plane; margins entire; mycelium sulphur yellow (15) at center, white at edge; texture floccose; sporulation absent; soluble pigments absent; exudates clear droplets; reverse saffron (10). MEA 25 °C, 7 d: Colonies moderately deep, plane; margins entire; mycelium white and sulphur yellow (15); texture floccose; sporulation sparse; conidia *en masse* white to greyish yellow-green (68); soluble pigments absent; exudates clear droplets; reverse saffron (10). YES 25 °C, 7 d: Colonies moderately deep, plane; margins entire; mycelium white and buff (45); texture floccose; sporulation sparse; conidia *en masse* white to greyish yellow-green (68); soluble pigments absent; exudates clear droplets; reverse saffron (10). DG18 25 °C, 7 d: Colonies moderately deep, plane; margins entire; mycelium white; texture floccose; sporulation absent; soluble pigments absent; exudates absent; reverse cream white. OA 25 °C, 7 d: Colonies moderately deep, plane; margins entire; mycelium white and sulphur yellow (15); texture floccose; sporulation absent; soluble pigments absent; exudates clear droplets; reverse white. CREA 25 °C, 7 d: No growth.

#### Micromorphology.

Conidiophores monoverticillate, stipes smooth, 50–150 × 3–4.5 μm, phialides 3–9, flask shaped, 9–12 × 3–3.5 μm; conidia finely rough, ellipsoidal, 2–3 × 1.5–2 μm. Ascomata not observed.

#### Notes.

*Talaromyces resedanus* grows restrictedly on DG18 and does not grow on CREA, two features shared with other taxa in section Subinflati. The monoverticillate conidiophores can differentiate *T. resedanus* from all reported section Subinflati species. *Talaromyces aerugineus*, *T. flavus*, *T. intermedius*, *T. rotundus*, *T. tardifaciens* also produce monoverticillate conidiophores, all of them except *T. aerugineus* can produce ascospores. *Talaromyces aerugineus* differs from *T. resedanus* by its shorter conidiophores (10–20 × 2.5–5 μm) and large, globose to ellipsoidal conidia (3–8.5 × 2.5–5 μm).

This species was introduced as *Penicillium resedanum* (McLennan et al., 1954). Yilmaz et al. (2014) listed it as doubtful species because the ex-type culture CBS 181.71 was not viable at that time. We requested the lyophilized culture of CBS 181.71 and CBS 184.90 deposited in nitrogen, and successfully resurrected them. The concatenated alignment and the single gene phylogenies proved its assignment in section Subinflati. *Talaromyces omanensis* described by Halo et al. (2019) shares ITS, *BenA* and *CaM* (all 100% similarity) sequences with *T. resedanus* CBS 184.90, have 99.7% (576/578), 98.3% (357/363), 98.8% (487/493) similarity with *T. resedanus* CBS 181.71^T^. Its type culture SQUCC 13153 showed good sporulation on CYA and MEA and thus displayed green colony. The monoverticillate conidiophores, size and shape of stipes, phialides and conidia of *T. omanensis* resemble those of *T. resedanus*, except that conidiophores of *T. omanensis* are rough under scanning electron microscope (SEM) (Halo et al. 2019). The photo plate of *T. omanensis* showed smooth conidiophores under microscope. Based on the molecular and morphological similarity, we considered *T. omanensis* a synonym of *T. resedanus*.

**Figure 10.**
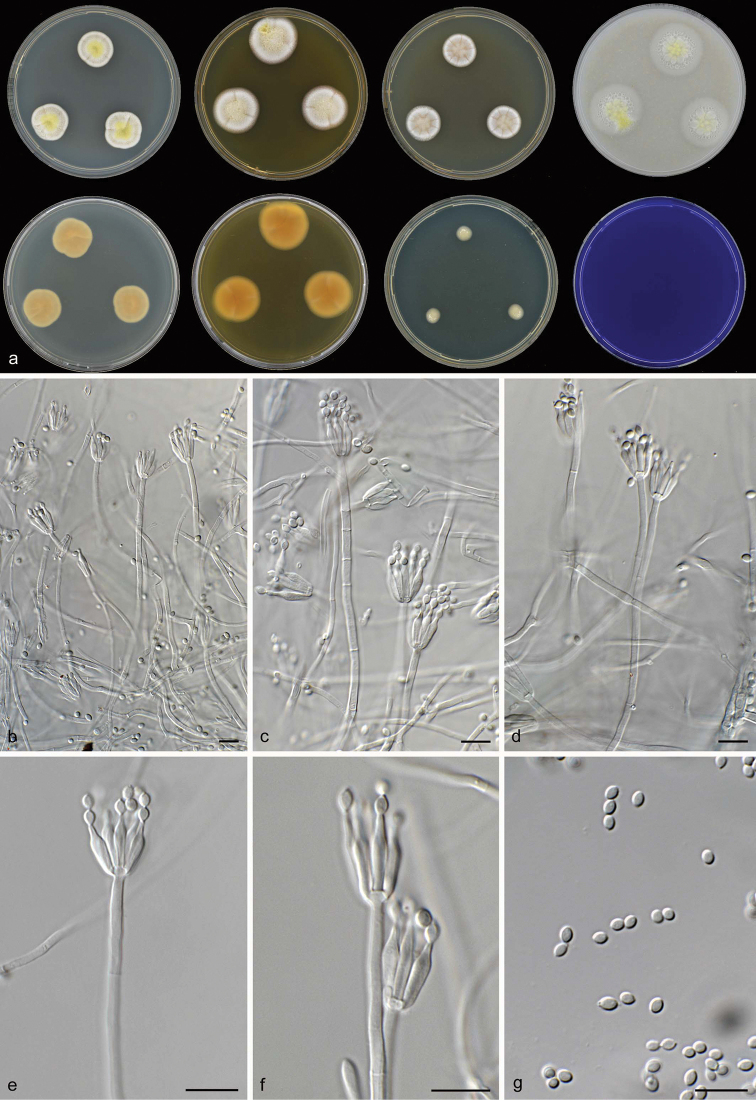
*Talaromyces resedanus* CBS 181.71^T^**a** colonies from left to right (top row) CYA, MEA, YES and OA; (bottom row) CYA reverse, MEA reverse, DG 18 and CREA**b–g** conidiophores and conidia. Scale bars: 10 μm (**b–g**).

### Talaromyces rufus

Taxon classificationFungiEurotialesAspergillaceae

B.D. Sun, A.J. Chen, Houbraken & Samson sp. nov.

A865909C-8672-52AD-BE6E-58C6E86A2122

833133

Fig. 11

#### Typus.

**China**, Yunnan, soil, 2009, isolated by T.S. Zhou, Holotype CBS H-22832, culture ex-holotype CBS 141834 = DTO 349-D7 = CGMCC 3.13203.

#### Additional material examined.

Korea, soil, 2013, isolated by J. Houbraken, culture DTO 274-C5.

#### ITS barcode.

MN864272. Alternative identification markers: *BenA* = MN863341, *CaM* = MN863318, *RPB2* = MN863331.

#### Diagnosis.

This species produces red, determinate synnemata and ellipsoidal, spiny ascospores measuring 5–6 × 4–5 μm.

#### In.

**Talaromyces section Talaromyces**

#### Colony diam, 7 d (mm).

CYA 12–16; CYA 30 °C 18–20; CYA 37 °C 15–16; MEA 37–38; MEA 30 °C 50–51; OA 38–40; YES 26–27; CREA Weak growth; CYAS No growth; DG18 9–13.

#### Colony characters.

CYA 25 °C, 7 d: Colonies deep, plane; margins entire; mycelium white and scarlet (5); texture floccose; sporulation sparse; conidia *en masse* greyish yellow-green (68); soluble pigments scarlet (5); exudates absent; reverse scarlet (5). MEA 25 °C, 7 d: Colonies moderately deep, plane; margins entire; mycelium white and scarlet (5); texture floccose; sporulation sparse; conidia *en masse* greyish yellow-green (68); soluble pigments scarlet (5); exudates absent; reverse scarlet (5). YES 25 °C, 7 d: Colonies moderately deep, raised at center, plane; margins entire; mycelium white and scarlet (5); texture floccose; sporulation absent; soluble pigments absent; exudates scarlet (5) droplets; reverse scarlet (5) at center, fading into peach (4). DG18 25 °C, 7 d: Colonies moderately deep, plane; margins entire; mycelium white; texture smooth and sticky; sporulation absent; soluble pigments absent; exudates absent; reverse cream white. OA 25 °C, 7 d: Colonies low, plane; margins entire; mycelium white and scarlet (5); texture floccose; sporulation absent; soluble pigments scarlet (5); exudates absent; reverse scarlet (5). Ascomata present. CREA 25 °C, 7 d: Acid production absent.

#### Micromorphology.

Conidiophores solitary and monoverticillate; stipes smooth, 5–30 × 2.5–3 μm; phialides1–4, acerose, 10–12 × 3–4 μm; conidia smooth, ellipsoidal to fusiform, 2.5–4.5 × 2–3 μm. Ascomata maturing within 2–3 wk on OA, subglobose to ellipsoildal, 350–600 × 200–350 μm, yellow, ascospores ellipsoidal, spiny, 5–6 × 4–5 μm.

#### Notes.

*Talaromyces rufus* is characterized by its red determinate synnemata on all tested media except DG18, CYAS and CREA. According to Yilmaz et al. (2014), twelve *Talaromyces* species produce determinate or indeterminate synnemata, but *T. rufus* can be easily distinguished from them by its red synnemata. Phylogenetically, *T. rufus* is related to *T. macrosporus*; however, *T. macrosporus* produces broadly ellipsoidal ascospores and does not produce synnemata.

#### Etymology.

Latin, *rufus*, refers to its red synnemata.

**Figure 11.**
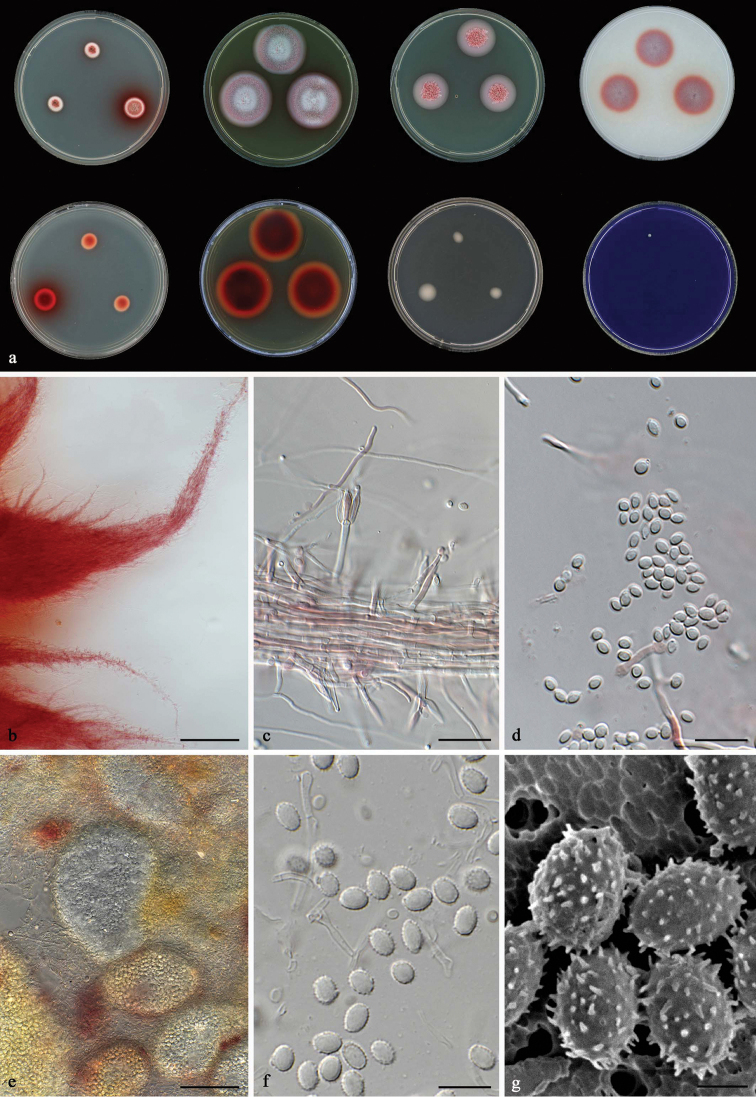
*Talaromyces rufus* CBS 141834^T^**a** colonies from left to right (top row) CYA, MEA, YES and OA; (bottom row) CYA reverse, MEA reverse, DG 18 and CREA**b** synnemata on MEA after 1 wk incubation **c, d** conidiophores and conidia **e** ascomata **f, g** ascospores. Scale bars: 200 μm (**b**), 10 μm (**c, d, f**), 50 μm (**e**), 2 μm (**g**).

## Discussion

Previous studies showed that the genus *Talaromyces* comprises seven sections (Yilmaz et al. 2014). In this study, a comprehensive isolation of soil samples was carried out in China; one new section and six new species were described using a polyphasic approach. Talaromyces section Tenues is newly introduced and contains one species. In our phylogenetic analysis, this section is sister to sections *Talaromyces* and *Helici*, though statistical support is lacking. Houbraken et al. (2020) studied the relationships within the *Eurotiales* and confidently showed that the section is sister to *Purpurei* and *Trachyspermi*. section Tenues is morphologically characterized by restricted growth on CYA, YES and DG18, slightly faster growth on MEA and OA and no growth on CREA. Based on these phenotypic characteristics, section Tenues species resemble section Trachyspermi species, but the species in section Trachyspermi are likely to produce abundant red pigments (Yilmaz et al. 2014), while *Talaromyces tenuis* doesn’t. *Talaromyces tenuis* also produces thinner conidiophores, and it is interesting to find out whether this character is shared by other species that will be described in this section in the future.

Three of our new species fall into section Talaromyces. This section was first introduced for species that produce yellow ascomata, which can occasionally be white, creamish, pinkish or reddish, and have yellow ascospores (Stolk and Samson 1972). Nowadays, this section is not limited to sexual species, but it still contains the largest number and highest ratio of sexual reproducing species in this genus. Among three new species, *T. brevis* and *T. rufus* produce yellow, spiny ascospores, the ascospores of *T. brevis* are smaller compared to its close relatives *T. liani*, and *T. rufus* can be easily distinguished by its red synnemata. *Talaromyces aspriconidius* is characterized by its strikingly roughened, globose conidia, but ascospores were not observed after long incubation.

*Talaromyces albisclerotius* is classified in section Trachyspermi. Species in section Trachyspermi show restricted growth on CYA, YES and DG18, grow slightly faster on MEA, and do not, or poorly grow, on CREA. Conidiophores are generally biverticillate and some species produce creamish white or yellow ascomata (Yilmaz et al. 2014). The morphology of *T. albisclerotius* matches these characters well; however, *T. albisclerotius* produces white sclerotia and does not produce ascomata and ascospores. Yilmaz et al. (2016c) speculated that *Talaromyces* species with no known sexual stage may actually be heterothallic. They successfully induced the sexual reproductive structures in *T. amestolkiae*, which was formerly described as an asexual taxon with black sclerotia. Further study is needed on *T. albisclerotius* to complete this hypothesis.

*Talaromyces guizhouensis* and *T. resedanus* belong to section Subinflati. This section previously contained two morphologically distinct species *T. palmae* and *T. subinflatus*. *Talaromyces palmae* produces short, biverticillate conidiophores and indeterminate synnemata, and *T. subinflatus* produces longer conidiophores, and grows more restrictedly on all tested media (Yilmaz et al. 2014). *Talaromyces tzapotlensis* was described by Peterson and Jurjević (2017), it grows well on CREA and DG18. *Talaromyces omanensis* was considered as a synonym of *T. resedanus* based on molecular and morphological similarity. The growth rate of *T. guizhouensis* falls somewhere in between; it is phylogenetically close to *T. subinflatus* and *T. tzapotlensis*. *Talaromyces resedanus* is the only monoverticillate species in this section.

*Talaromyces* species have a worldwide distribution and are isolated from a wide range of substrates. Soil is their main habitat, but new species were also isolated from indoor air, dust, clinical samples, plants, seed, leaf litter, honey, pollen and stingless bee nests (Sang et al. 2013; Visagie et al. 2014; Chen et al. 2016; Wang QM et al. 2016; Yilmaz et al. 2016a; Guevara-Suarez et al. 2017; Peterson and Jurjević 2017; Barbosa et al. 2018; Crous et al. 2018; Su and Niu 2018; Rodríguez-Andrade et al. 2019). These studies expanded our knowledge on the substrates where *Talaromyces* species can occur, but on the other hand demonstrated the complicated ecological function of this genus. In this study, one new section and six new species were identified from soil in China. Further research will focus on the *Talaromyces* diversity from a wide range of substrates.

## Supplementary Material

XML Treatment for Talaromyces section Tenues

XML Treatment for Talaromyces tenuis

XML Treatment for Talaromyces albisclerotius

XML Treatment for Talaromyces aspriconidius

XML Treatment for Talaromyces brevis

XML Treatment for Talaromyces guizhouensis

XML Treatment for Talaromyces resedanus

XML Treatment for Talaromyces rufus

## Supplementary materials

Supplementary material 1 Phylogeny of ITS for species classified in Talaromyces section Talaromyces Data type phylogenetic Explanation note Branches with values more than 1 pp and 95% bs are thickened. *Talaromyces dendriticus* was chosen as outgroup. https://binary.pensoft.net/file/429701This dataset is made available under the Open Database License (http://opendatacommons.org/licenses/odbl/1.0/). The Open Database License (ODbL) is a license agreement intended to allow users to freely share, modify, and use this Dataset while maintaining this same freedom for others, provided that the original source and author(s) are credited.Bing-Da Sun, Amanda J. Chen, Jos Houbraken, Jens C. Frisvad, Wen-Ping Wu, Hai-Lei Wei, Yu-Guang Zhou, Xian-Zhi Jiang, Robert A. Samson

Supplementary material 2 Phylogeny of *CaM* for species classified in Talaromyces section Talaromyces Data type phylogenetic Explanation note Branches with values more than 1 pp and 95% bs are thickened. *Talaromyces dendriticus* was chosen as outgroup. https://binary.pensoft.net/file/429702This dataset is made available under the Open Database License (http://opendatacommons.org/licenses/odbl/1.0/). The Open Database License (ODbL) is a license agreement intended to allow users to freely share, modify, and use this Dataset while maintaining this same freedom for others, provided that the original source and author(s) are credited.Bing-Da Sun, Amanda J. Chen, Jos Houbraken, Jens C. Frisvad, Wen-Ping Wu, Hai-Lei Wei, Yu-Guang Zhou, Xian-Zhi Jiang, Robert A. Samson

Supplementary material 3 Phylogeny of *RPB2* for species classified in Talaromyces section Talaromyces Data type phylogenetic Explanation note Branches with values more than 1 pp and 95% bs are thickened. *Talaromyces dendriticus* was chosen as outgroup. https://binary.pensoft.net/file/429703This dataset is made available under the Open Database License (http://opendatacommons.org/licenses/odbl/1.0/). The Open Database License (ODbL) is a license agreement intended to allow users to freely share, modify, and use this Dataset while maintaining this same freedom for others, provided that the original source and author(s) are credited.Bing-Da Sun, Amanda J. Chen, Jos Houbraken, Jens C. Frisvad, Wen-Ping Wu, Hai-Lei Wei, Yu-Guang Zhou, Xian-Zhi Jiang, Robert A. Samson

Supplementary material 4 Phylogeny of ITS for species classified in Talaromyces section Trachyspermi Data type phylogenetic Explanation note Branches with values more than 1 pp and 95% bs are thickened. *Talaromyces purpurogenus* was chosen as outgroup. https://binary.pensoft.net/file/429704This dataset is made available under the Open Database License (http://opendatacommons.org/licenses/odbl/1.0/). The Open Database License (ODbL) is a license agreement intended to allow users to freely share, modify, and use this Dataset while maintaining this same freedom for others, provided that the original source and author(s) are credited.Bing-Da Sun, Amanda J. Chen, Jos Houbraken, Jens C. Frisvad, Wen-Ping Wu, Hai-Lei Wei, Yu-Guang Zhou, Xian-Zhi Jiang, Robert A. Samson

Supplementary material 5 Phylogeny of *CaM* for species classified in Talaromyces section Trachyspermi Data type phylogenetic Explanation note Branches with values more than 1 pp and 95% bs are thickened. *Talaromyces dendriticus* was chosen as outgroup. https://binary.pensoft.net/file/429705This dataset is made available under the Open Database License (http://opendatacommons.org/licenses/odbl/1.0/). The Open Database License (ODbL) is a license agreement intended to allow users to freely share, modify, and use this Dataset while maintaining this same freedom for others, provided that the original source and author(s) are credited.Bing-Da Sun, Amanda J. Chen, Jos Houbraken, Jens C. Frisvad, Wen-Ping Wu, Hai-Lei Wei, Yu-Guang Zhou, Xian-Zhi Jiang, Robert A. Samson

Supplementary material 6 Phylogeny of *RPB2* for species classified in Talaromyces section Trachyspermi Data type phylogenetic Explanation note Branches with values more than 1 pp and 95% bs are thickened. *Talaromyces dendriticus* was chosen as outgroup. https://binary.pensoft.net/file/429706This dataset is made available under the Open Database License (http://opendatacommons.org/licenses/odbl/1.0/). The Open Database License (ODbL) is a license agreement intended to allow users to freely share, modify, and use this Dataset while maintaining this same freedom for others, provided that the original source and author(s) are credited.Bing-Da Sun, Amanda J. Chen, Jos Houbraken, Jens C. Frisvad, Wen-Ping Wu, Hai-Lei Wei, Yu-Guang Zhou, Xian-Zhi Jiang, Robert A. Samson

Supplementary material 7 Phylogeny of ITS for species classified in Talaromyces section Subinflati Data type phylogenetic Explanation note Branches with values more than 1 pp and 95% bs are thickened. *Talaromyces dendriticus* was chosen as outgroup. https://binary.pensoft.net/file/429707This dataset is made available under the Open Database License (http://opendatacommons.org/licenses/odbl/1.0/). The Open Database License (ODbL) is a license agreement intended to allow users to freely share, modify, and use this Dataset while maintaining this same freedom for others, provided that the original source and author(s) are credited.Bing-Da Sun, Amanda J. Chen, Jos Houbraken, Jens C. Frisvad, Wen-Ping Wu, Hai-Lei Wei, Yu-Guang Zhou, Xian-Zhi Jiang, Robert A. Samson

Supplementary material 8 Phylogeny of *CaM* for species classified in Talaromyces section Subinflati Data type phylogenetic Explanation note Branches with values more than 1 pp and 95% bs are thickened. *Talaromyces dendriticus* was chosen as outgroup. https://binary.pensoft.net/file/429708This dataset is made available under the Open Database License (http://opendatacommons.org/licenses/odbl/1.0/). The Open Database License (ODbL) is a license agreement intended to allow users to freely share, modify, and use this Dataset while maintaining this same freedom for others, provided that the original source and author(s) are credited.Bing-Da Sun, Amanda J. Chen, Jos Houbraken, Jens C. Frisvad, Wen-Ping Wu, Hai-Lei Wei, Yu-Guang Zhou, Xian-Zhi Jiang, Robert A. Samson

Supplementary material 9 Phylogeny of *RPB2* for species classified in Talaromyces section Subinflati Data type phylogenetic Explanation note Branches with values more than 1 pp and 95% bs are thickened. *Talaromyces dendriticus* was chosen as outgroup. https://binary.pensoft.net/file/429709This dataset is made available under the Open Database License (http://opendatacommons.org/licenses/odbl/1.0/). The Open Database License (ODbL) is a license agreement intended to allow users to freely share, modify, and use this Dataset while maintaining this same freedom for others, provided that the original source and author(s) are credited.Bing-Da Sun, Amanda J. Chen, Jos Houbraken, Jens C. Frisvad, Wen-Ping Wu, Hai-Lei Wei, Yu-Guang Zhou, Xian-Zhi Jiang, Robert A. Samson
