# Supplementary figures and images for: New section and species in Talaromyces
Source: MycoKeys. 2020 Jul 7;68:75–113. doi: 10.3897/mycokeys.68.52092 (PMC7360636; doi:10.3897/mycokeys.68.52092)

# Sect. *Talaromyces*

## ITS

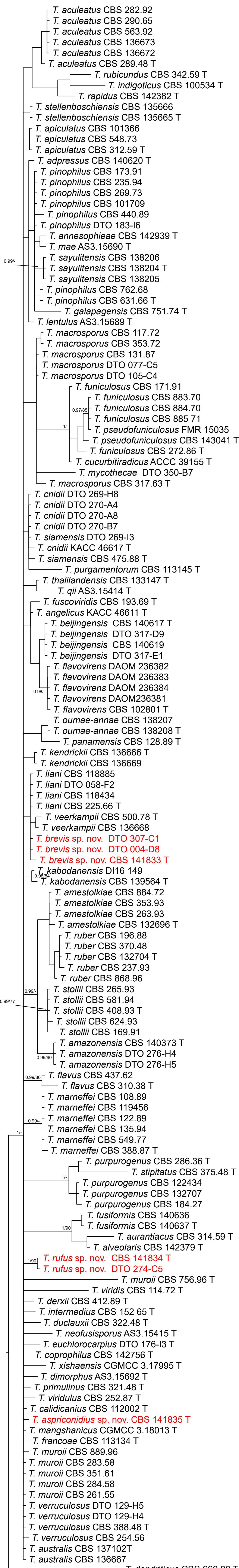

0.2

Supplement: Supplementary material 1 — Phylogeny of ITS for species classified in Talaromyces section Talaromyces [file mycokeys-68-075-s001.pdf]

*Sect. Talaromyces*  
**CaM**

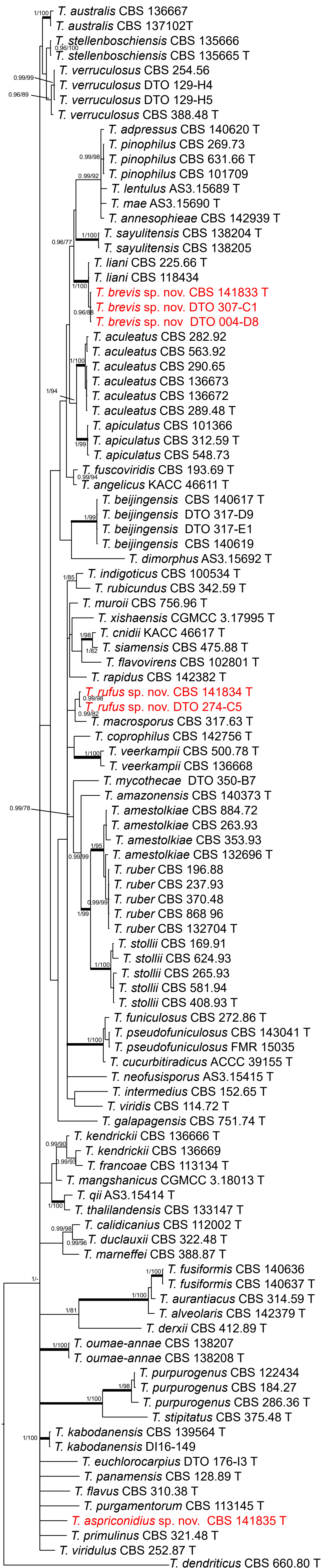

0.1

Supplement: Supplementary material 2 — Phylogeny of CaM for species classified in Talaromyces section Talaromyces [file mycokeys-68-075-s002.pdf]

Sect. *Talaromyces*  
*RPB2*

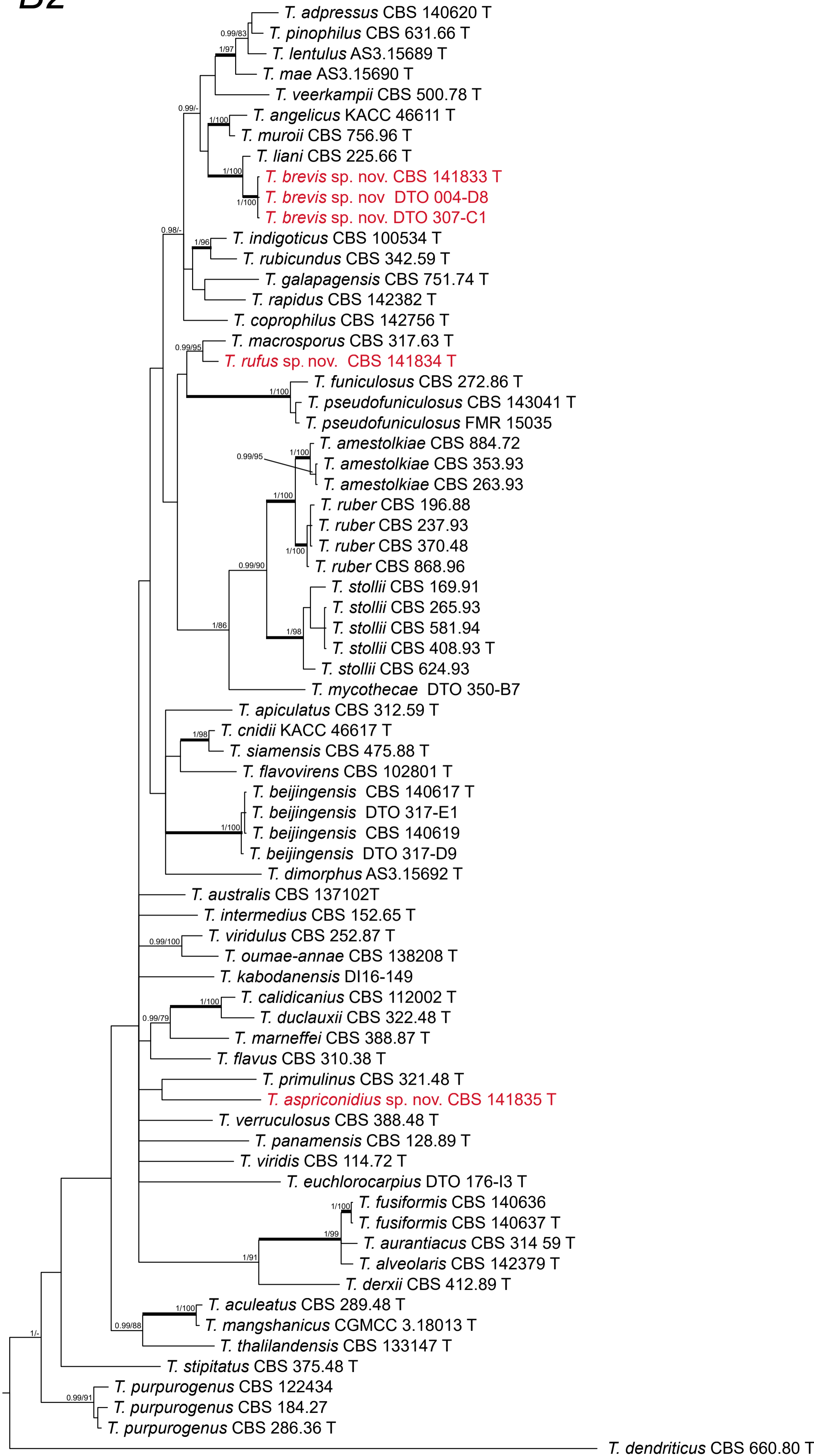

Supplement: Supplementary material 3 — Phylogeny of RPB2 for species classified in Talaromyces section Talaromyces [file mycokeys-68-075-s003.pdf]

# Sect. *Trachyspermi*

## ITS

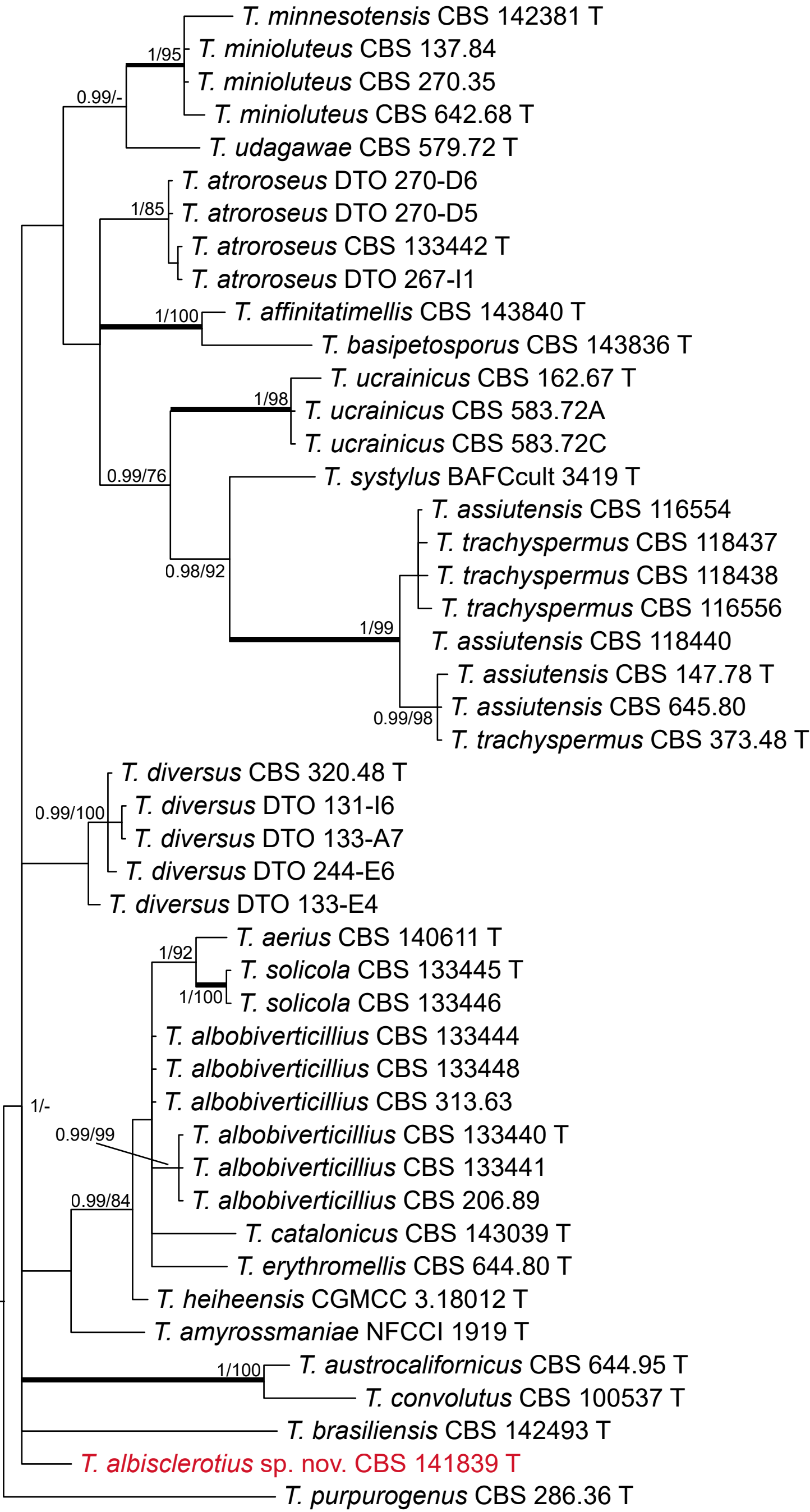

0.05

Supplement: Supplementary material 4 — Phylogeny of ITS for species classified in Talaromyces section Trachyspermi [file mycokeys-68-075-s004.pdf]

# Sect. *Trachyspermi*

## CaM

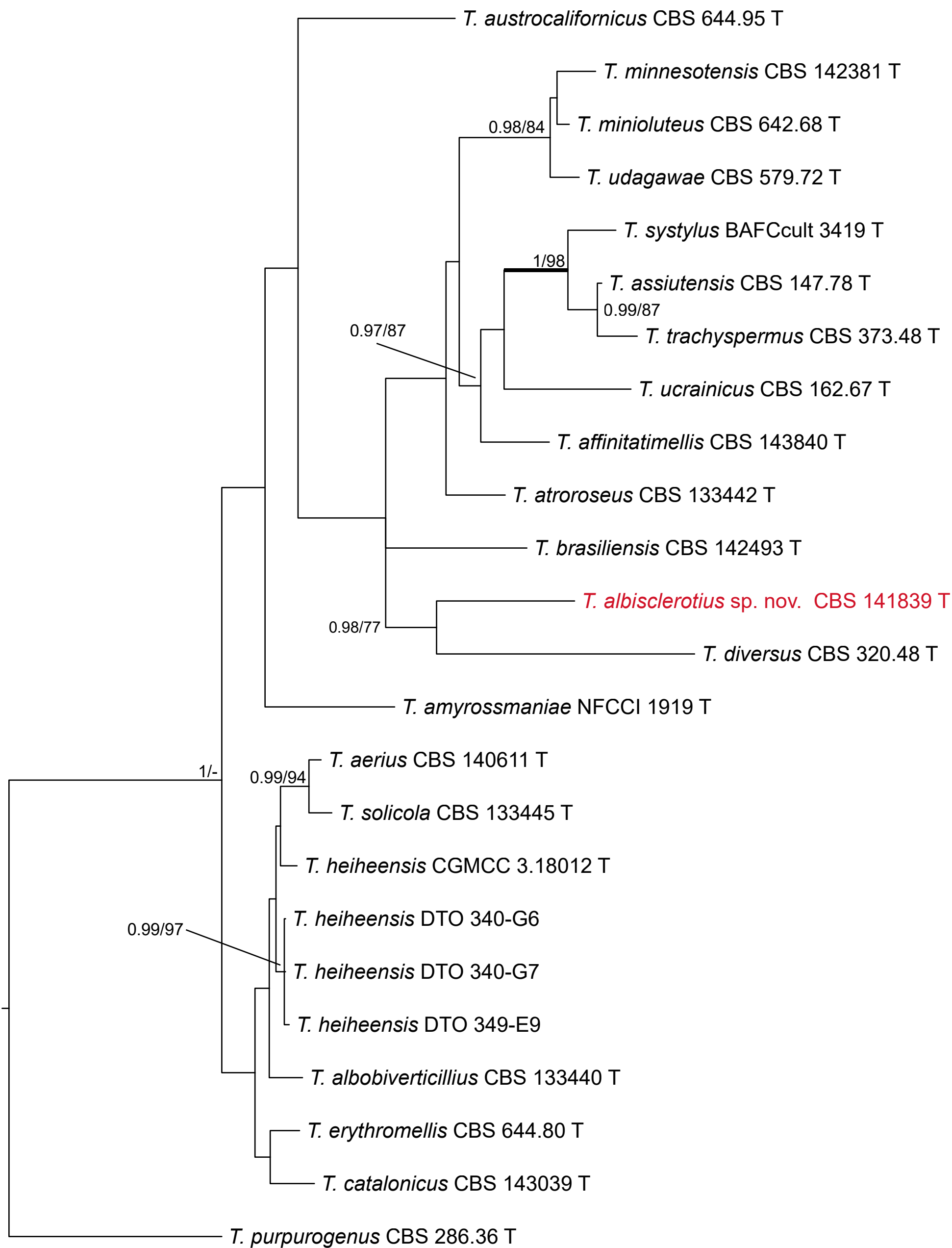

0.07

Supplement: Supplementary material 5 — Phylogeny of CaM for species classified in Talaromyces section Trachyspermi [file mycokeys-68-075-s005.pdf]

# Sect. *Trachyspermi*

## RPB2

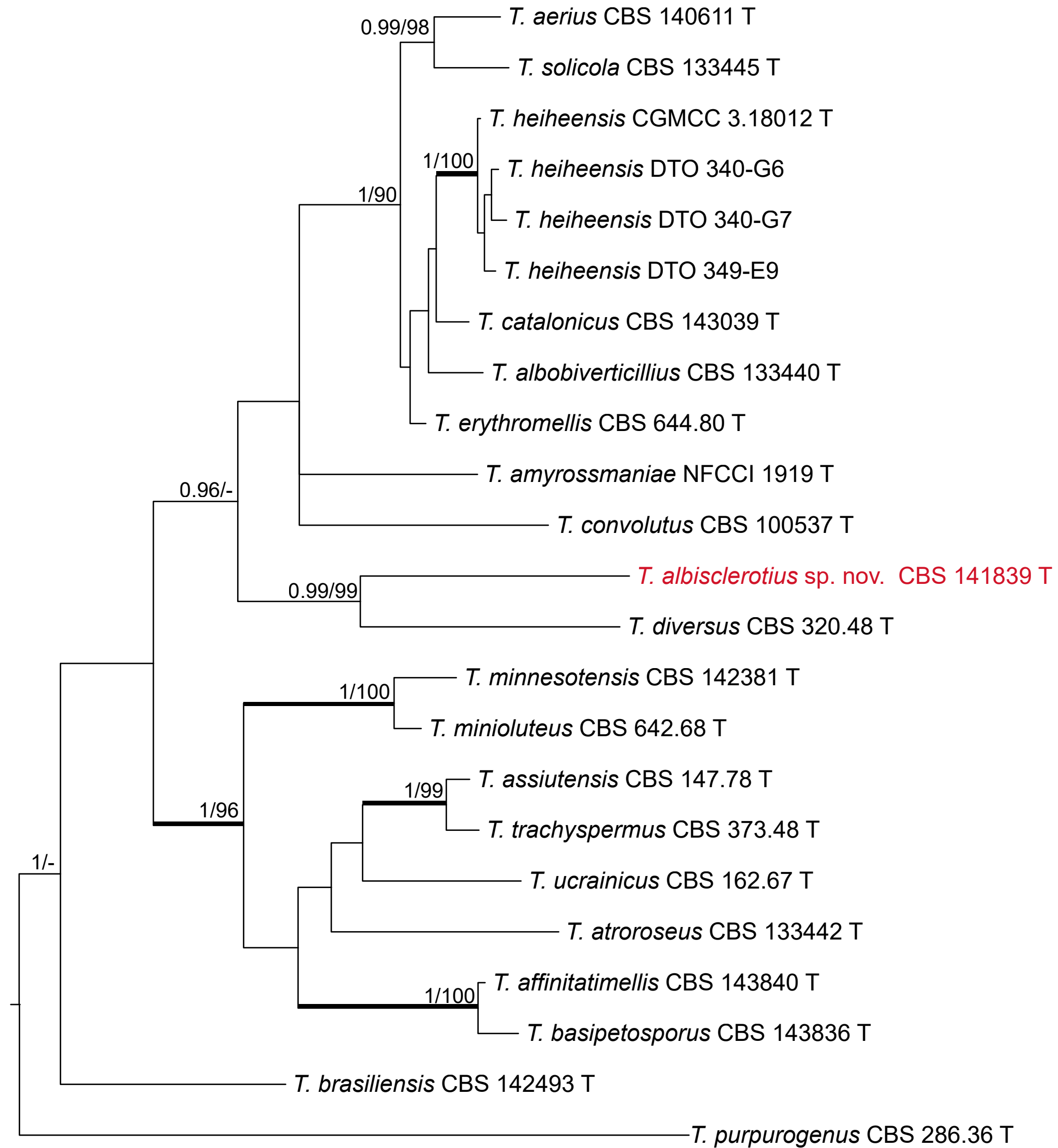

Supplement: Supplementary material 6 — Phylogeny of RPB2 for species classified in Talaromyces section Trachyspermi [file mycokeys-68-075-s006.pdf]

Sect. *Subinflati* ITS

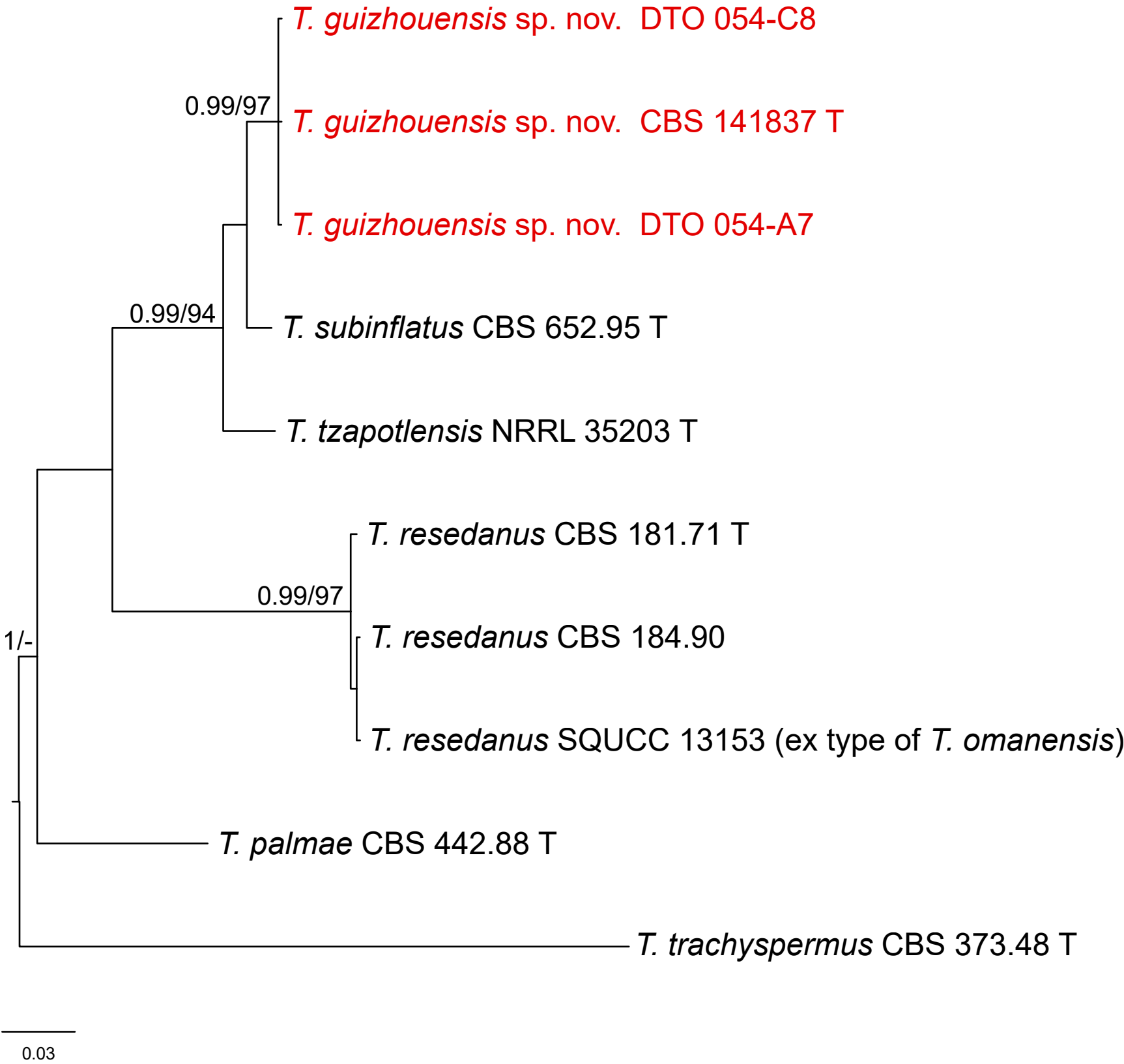

Supplement: Supplementary material 7 — Phylogeny of ITS for species classified in Talaromyces section Subinflati [file mycokeys-68-075-s007.pdf]

Sect. *Subinflati* CaM

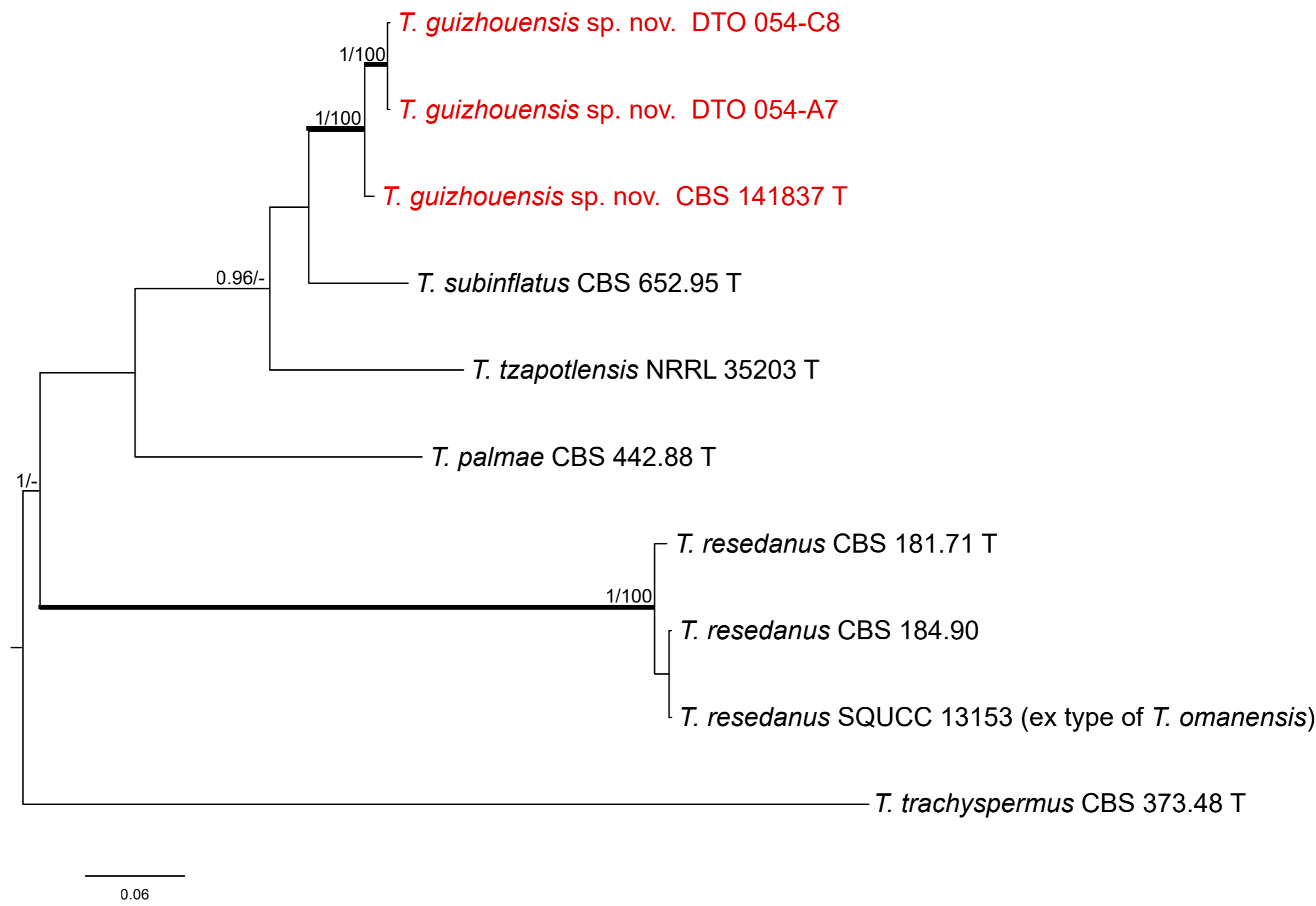

Supplement: Supplementary material 8 — Phylogeny of CaM for species classified in Talaromyces section Subinflati [file mycokeys-68-075-s008.pdf]

*Sect. Subinflati RPB2*

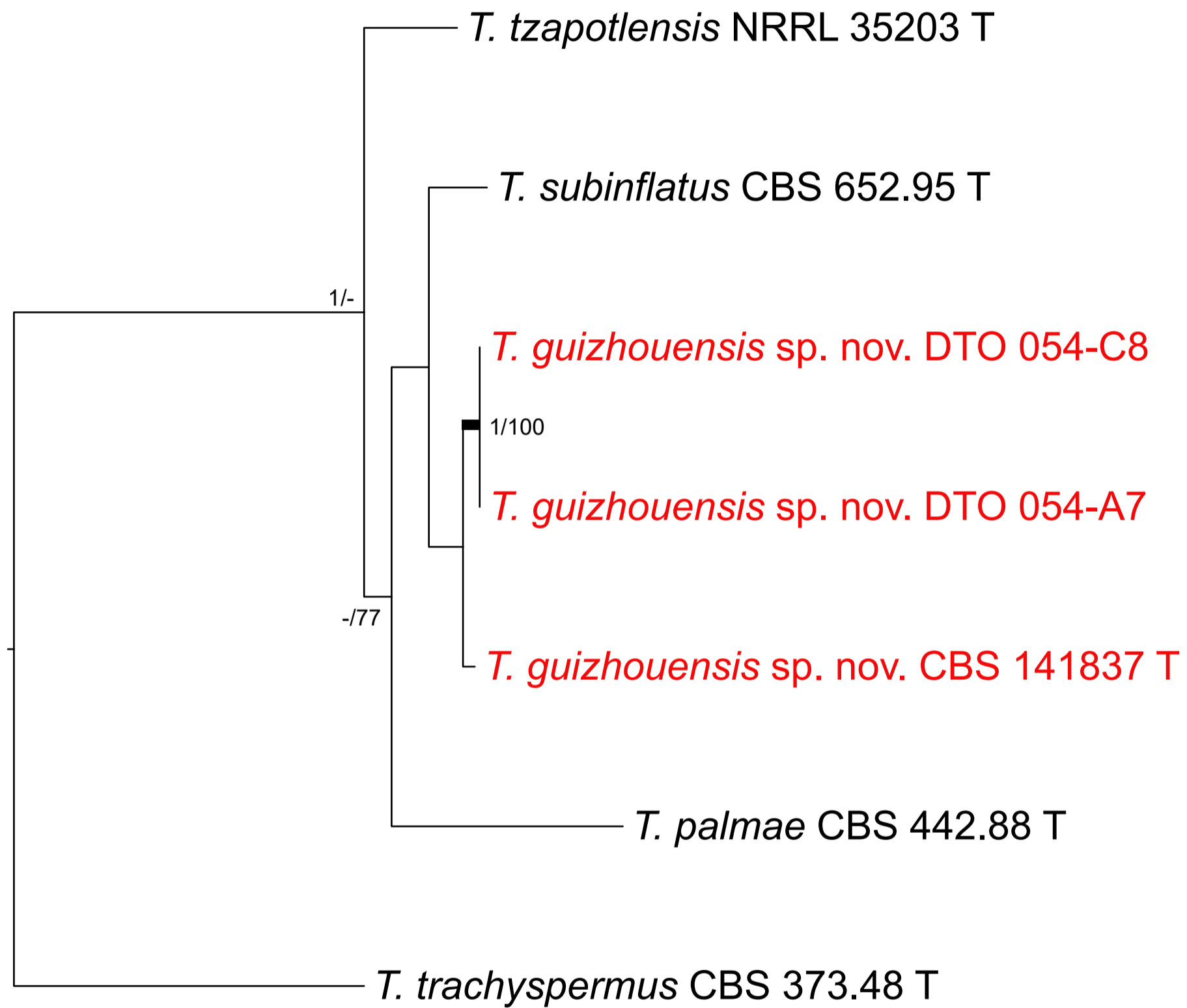

0.04

Supplement: Supplementary material 9 — Phylogeny of RPB2 for species classified in Talaromyces section Subinflati [file mycokeys-68-075-s009.pdf]
